# Elucidation of the Active Form and Reaction Mechanism in Human Asparaginase Type III Using Multiscale Simulations

**DOI:** 10.1021/acs.jcim.3c00900

**Published:** 2023-08-28

**Authors:** Milorad Andjelkovic, Kirill Zinovjev, Carlos Alberto Ramos-Guzmán, Jose Javier Ruiz- Pernía, Iñaki Tuñón

**Affiliations:** † Departamento de Química Física, Universidad de Valencia, 46100 Burjassot, Spain; ‡ Instituto de Materiales Avanzados, Universidad Jaume I, 12071 Castelló, Spain

## Abstract

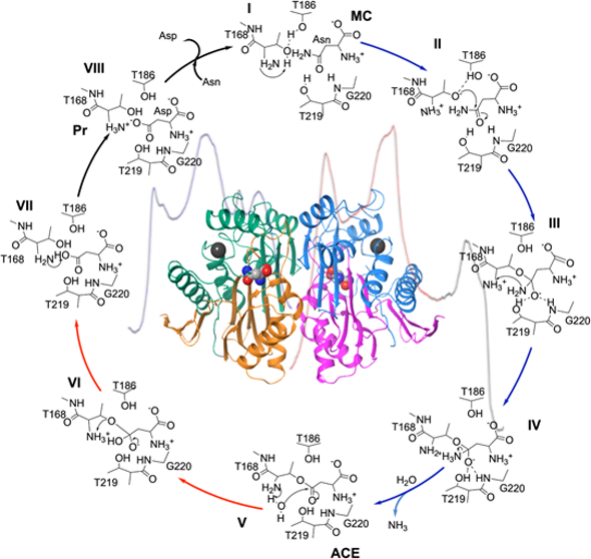

l-asparaginases catalyze the asparagine hydrolysis to aspartate. These enzymes play an important role in the treatment of acute lymphoblastic leukemia because these cells are unable to produce their own asparagine. Due to the immunogenic response and various side effects of enzymes of bacterial origin, many attempts have been made to replace these enzymes with mammalian enzymes such as human asparaginase type III (hASNaseIII). This study investigates the reaction mechanism of hASNaseIII through molecular dynamics simulations, quantum mechanics/molecular mechanics methods, and free energy calculations. Our simulations reveal that the dimeric form of the enzyme plays a vital role in stabilizing the substrate in the active site, despite the active site residues coming from a single protomer. Protomer–protomer interactions are essential to keep the enzyme in an active conformation. Our study of the reaction mechanism indicates that the self-cleavage process that generates an N-terminal residue (Thr168) is required to activate the enzyme. This residue acts as the nucleophile, attacking the electrophilic carbon of the substrate after a proton transfer from its hydroxyl group to the N-terminal amino group. The reaction mechanism proceeds with the formation of an acyl-enzyme complex and its hydrolysis, which turns out to be the rate-determining step. Our proposal of the enzymatic mechanism sheds light on the role of different active site residues and rationalizes the studies on mutations. The insights provided here about hASNaseIII activity could contribute to the comprehension of the disparities among different ASNases and might even guide the design of new variants with improved properties for acute lymphoblastic leukemia treatment.

## 1 Introduction

l-Asparaginases (L-ASNases or just ASNases, E.C. 3.5.1.1) are enzymes hydrolyzing l-asparagine (Asn) to l-aspartic acid (Asp) and ammonia.^1^ Based on the protein sequence, biochemical properties, and crystallographic data, ASNases have been classified into three classes (1, 2, and 3) containing five subclasses or types (from I to V).^2^ Apart from being widely used in food manufacturing, reducing acrylamide in food,^3^ the main use of ASNases is in the treatment of various types of cancer, such as acute myeloid leukemia,^4^ lymphosarcoma,^5^ several types of non-Hodgkin’s lymphoma,^6^ and Acute Lymphoblastic Leukemia (ALL).^5,7^ ALL cells are dependent on the extracellular pool of Asn amino acid because they are not able to express their own Asparagine Synthetase.^7^ Administration of l-ASNase to patients suffering from ALL hydrolyzes asparagine present in blood, depriving the leukemic cells of circulating asparagine and triggering their apoptosis. Two different types of ASNases are being used nowadays in the treatment of ALL: *Escherichia coli* type II (EcASNaseII) and *Erwinia chrysanthemi* type II (ErASNaseII), better known under their trade names Elspar and Erwinaze. However, several side effects of these treatments associated with the bacterial nature of the enzymes, such as silent inactivation^8^ of the enzyme and hypersensitivity reactions,^9^ have been reported. Some attempts have been done to diminish these side effects, such as the PEGylation of EcASNaseII (FDA-approved drug with the trade name Oncaspar), which improved the half-life of the drug.^10^ However, no significant progress was observed in regard to the diminution of the immunogenetic response.^11^ In order to overcome these side effects due to the bacterial origin of the enzymes used in ALL treatments, several trials have been oriented toward their substitution by l-ASNases of mammalian origins, such as human l-ASNases (hASNaseIII)^12,13^ and guinea pig ASNase (gpASNaseIII).^14,15^ While several theoretical studies have been performed on the reaction mechanism of l-ASNase type I and II,^16−22^ to our knowledge, no research has yet been done on the mechanism of ASNasesIII.

**Figure 1:**
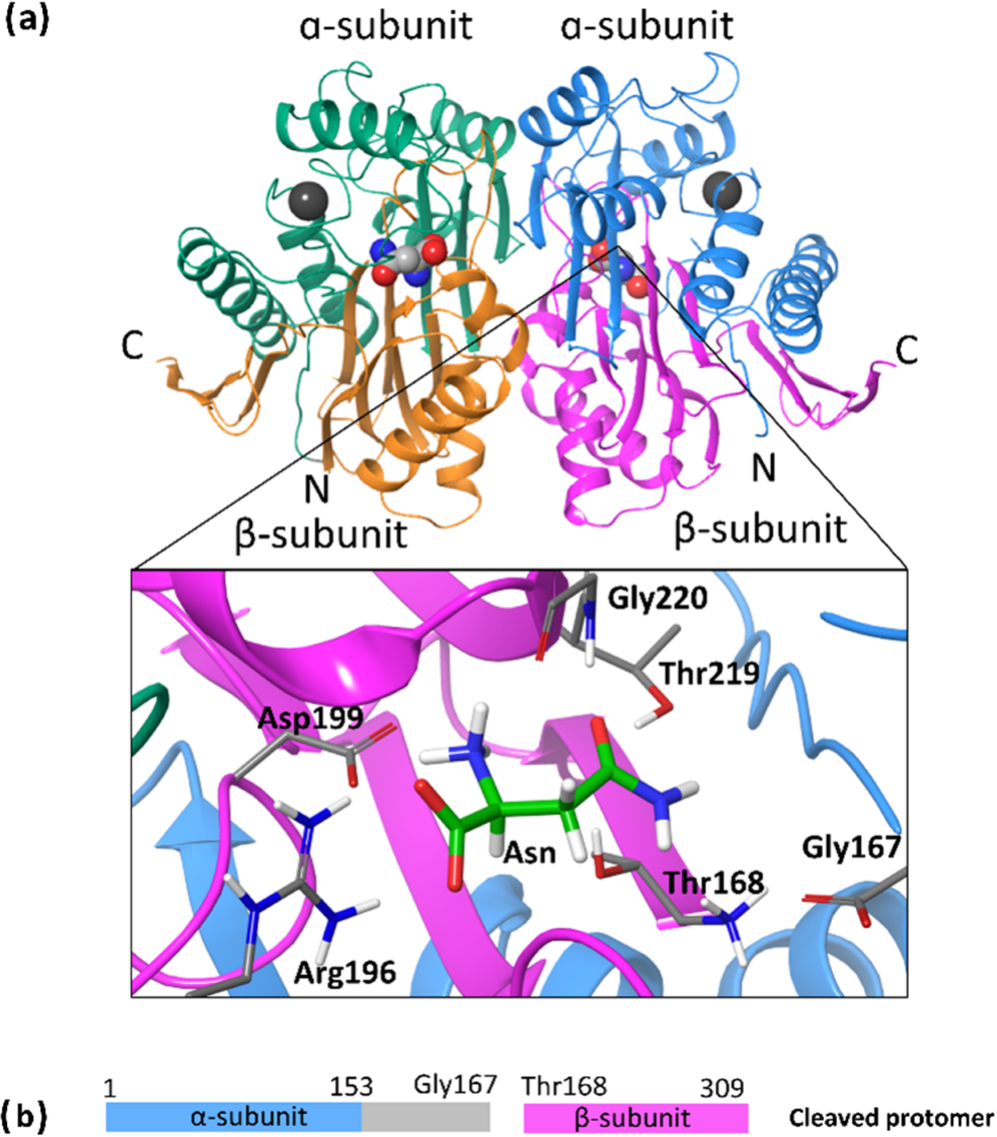
(a) Homodimer hASNaseIII structure (top) with the two substrates (balls representation) in the active sites and two sodium ions (dark gray balls) within the sodium-binding loops. Orange and green colors correspond to the subunits of protomer A, while blue and pink colors correspond to the protomer B. A close-up view of one active site of hASNaseIII is presented below. The carbon atoms of the substrate (Asn) are represented in light green color, while those of other residues are represented in gray. (b) Schematic representation of the sequence for the cleaved protomer B (corresponding to the formation of α and β subunits). Colors of the rectangles correspond to the colors of the subunits of protomer B given on the top, while the gray part corresponds to the residues spanning the flexible loop region (153–167).

ASNasesIII belongs to the N-terminal nucleophile (Ntn) hydrolases family. In this family, an enzymatic inactive precursor (the uncleaved protein) is activated after autoproteolytic cleavage.^23^ As a result of the cleavage reaction, the enzyme takes the structure of a αββα sandwich. In the case of hASNaseIII, the self-cleavage occurs after a flexible loop section (see Figure 1), between residues Gly167 and Thr168, resulting in the formation of the α and β subunits of each protomer of the dimeric structure and releasing Thr168 as an N-terminal residue that acts as the nucleophile during the ASNase activity. This is supported by the X-ray structure (4O0H) of the acyl-enzyme complex, where Thr168 is covalently bonded to the substrate.^13^ The structure of hASNaseIII includes a conserved sodium-binding loop, which is placed close to the active site in the α chain (see Figure 1).^1^ This loop (residues 55–65) was suggested to lock the catalytic Thr168 in place by means of a hydrogen bond between its terminal amino group and the loop residue Asn62.^1,24^ Even though the active site residues of Ntn enzymes originate from a single protomer, all known X-ray structures, including hASNaseIII, are dimers; it is unclear if dimerization is a requisite for the catalytic activity. Several polar interactions keep the asparagine substrate bound to the active site (see Figure 1). The α-carboxylic group of asparagine forms a bidentate salt bridge with the side chain of Arg196, while the amino group is stabilized by the side chain of Asp199 and the main-chain carbonyl group of Gly220. The side chain of asparagine is positioned near the side chain of Thr219 and the main-chain NH group of Gly220. These last interactions are important to maintain the orientation of the substrate side chain in the active site.

**Scheme 1:**
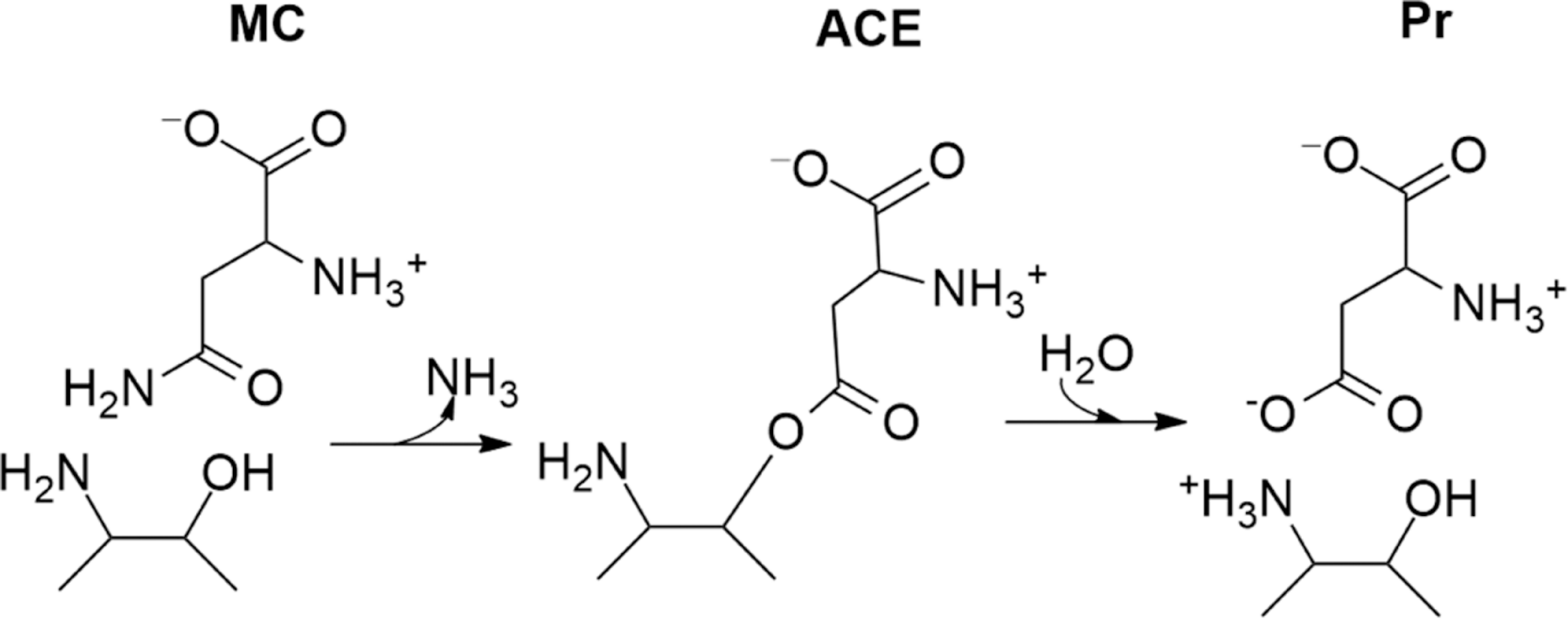
Catalytic Mechanism in Ntn-Hydrolase Enzymes Hydrolyzing Asparagine

The whole family of Ntn-hydrolases presents a similar three-dimensional fold, with the catalytic residues occupying equivalent sites.^25^ This similarity suggests a common reaction mechanism^26,27^ schematically depicted in Scheme 1. In this proposal, the hydroxyl oxygen of the N-terminal threonine is activated by means of proton abstraction. After activation, the nucleophilic Oγ attacks the Cγ atom of the substrate, forming an acyl-enzyme bond. This is followed by Cγ–Nδ bond breaking in the substrate and ammonia release. The covalent acyl-enzyme (ACE) intermediate is then hydrolyzed by a water molecule, forming the final product (Asp).

While the reaction mechanism of some Ntn enzymes has been studied,^26,28,29^ to our knowledge, no theoretical research has yet been done on the hASNaseIII. In this computational work, we address several issues that are relevant to understanding the activity of this enzyme. First, we carried out long Molecular Dynamics (MD) simulations to investigate the binding of the substrate in the active sites of the dimeric and monomeric forms of hASNaseIII, concluding that dimerization is a requirement for catalysis. Second, we investigated the role of the sodium-binding loops in stabilizing the nucleophile, Thr168, close to the substrate. We also performed free energy calculations to study the protonation state of this residue. The low free energy cost associated with its deprotonation enables the self-activation mechanism of Thr168 by means of a proton transfer to its N-terminal amino group. We finally used QM/MM calculations to determine the reaction mechanism for ASNase activity, unveiling the role of different residues and rationalizing the results of mutational studies. Our study may be useful to rationalize the differences observed in the activity of ASNases and to guide the design of new variants with improved properties for ALL treatment.

## 2 Methods

### 2.1 Preparation of the System

The Michaelis complex structure was built starting from the PDB structure 4O0H^13^ that corresponds to the homodimeric form (each monomer contains 309 amino acid residues) in the acyl-enzyme state. Since the loops spanned by residues 153–167 were not resolved, their structure was modeled using AlfaFold2.^30^ Afterward, the bond between residues 167 and 168 (and the corresponding one in the other monomer) was broken in order to simulate the cleaved protein state which is the active form needed for the ASNase activity. The covalent bond between the enzyme and the substrate was broken to form free asparagine in both active sites, using the Rotamers option of UCSF Chimera 1.14 interface^31^ to generate different initial conformations. The final conformation of the substrate in the Michaelis complex was selected by optimizing the interactions of the substrate with active site residues observed in the X-ray structure. All water molecules and sodium ions present in the X-ray structures were preserved. Missing H atoms were added using the Protein Preparation Wizard tool of Maestro,^32^ and PROPKA3.0^33^ was used to calculate the protonation states of titratable residues at pH 7.5. The Thr168N-terminal group was initially modeled as being protonated, and its p*K*_a_ was later evaluated, as explained below.

### 2.2 Classical Molecular Dynamics Simulations

All classical molecular dynamics (MD) simulations were performed using Amber18 GPU version of pmemd.^34,35^ Parameters for the substrate, free Asn in its zwitterionic form, were taken from Horn et al.,^36^ while standard amino acids were described using the ff14SB force field.^37^ The system was then immersed in a box of TIP3P^38^ water molecules, using the tleap tool from AmberTools18^39^ in such a way that protein-inhibitor atoms were at least 12 Å from the limits of the simulation box. After protonation, the total charge of the enzyme/substrate system was −10, which was compensated by the addition of 10 Na^+^ ions, resulting in a neutral system. After the system was minimized and equilibrated (see the SI for details), three replicas of 1 μs each were run for both the Michaelis complex and the apo form of the enzyme starting from the different initial velocities. Simulations were run in the NVT ensemble and using periodic boundary conditions. Temperature was maintained at 310 K using the Langevin thermostat, and the time step was set to 2 fs using SHAKE.^40^ Electrostatic interactions were calculated using particle-mesh Ewald,^41,42^ while the cutoff radius for nonelectrostatic interactions was set at 10.0 Å.

In order to calculate the p*K*_a_ of the N-terminal threonine, Thermodynamic Integration (TI) was employed as a free energy estimator along the alchemical transformation between the neutral and protonated forms (Figure S1) of this residue, as described by He et al.^43^ The parameters for the neutral N-terminal threonine were obtained using the nonstandard residue parametrization procedure implemented in Amber with the Antechamber^44^ program from the AmberTools18^39^ package, while the atomic charges were obtained using the restrained electrostatic potential (RESP)^45^ method at the HF/6-31G* level. The free energy was calculated by numerical integration of the derivative of the free energy over a coupling parameter (λ). The free energy cost associated with the release of ammonia from the active site was obtained using a similar procedure. The thermodynamic cycle followed is given in Figure S2, and more technical details are given in the SI section entitled Thermodynamic Integration.

### 2.3 QM/MM Calculations

To explore the free energy profiles associated with the chemical process, hybrid quantum mechanics/molecular mechanics (QM/MM) simulations were employed. To describe the QM subsystem, we used the B3LYP functional^46,47^ with D3 dispersion corrections^48^ and the 6-31+G* basis set. The B3LYP functional has been selected based on the previous works done on ASNases I and II,^16−18^ where this functional was found to provide energies in good agreement with the experimental data. Preliminary calculations used to explore different mechanistic proposals were made at the DFTB3/MM level.^49^ In general, the QM subsystem contained at least the whole substrate (Asn) and the N-terminal residue Thr168, placing the QM/MM boundary between C and Cα of the next residue (Val169). Other residues (water in the case of hydrolysis and regeneration steps) were included depending on the mechanism to be explored and are detailed in the corresponding figures. We tested different QM regions at the DFTB3 level, including also residues interacting with the substrate (Asp199, Arg196, Gly167), but no significant changes were found in the reaction free energy profiles (see Figure S3).

To explore the free energy profile of the reaction mechanism, the adaptive string method (ASM)^50,51^ developed in our research group was used. In this method, N replicas of the system (the string nodes) are defined along a putative reaction pathway connecting the product and reactant states in space defined by the set of collective variables (CVs). Nodes positions are evolved according to the free energy gradient while kept equidistant to ensure convergence to the Minimum Free Energy Path (MFEP). To ensure better convergence, Hamiltonian exchange between neighbor nodes is attempted every 50 steps. The CVs used at each stage of the mechanism are detailed below, but generally, we include all of the distances of bonds to be broken and formed. The root-mean-square deviation (RMSD) between the values of the CVs is used as an indicator of string convergence. After convergence (RMSD ∼ 0.1 amu^1/2^·Å for at least 2 ps), a path-CV (denoted as *s*) is defined to measure the position of the system along the MFEP. The obtained coordinate is then used as the reaction coordinate for umbrella sampling (US)^52^ calculations. The free energy profile was estimated using WHAM.^53,54^ The force constant values employed in US calculations were determined on-the-fly to ensure a homogeneous probability density distribution of the reaction coordinate.^51^ As stated above, mechanistic explorations with ASM were run first at the DFTB3/MM level. The best mechanistic proposals were then explored again at the B3LYPD3/MM level. B3LYPD3/MM molecular dynamics simulations were run using Amber18^39^ coupled to Gaussian16^55^ in the NVT ensemble, keeping the temperature at 310 K and using Langevin thermostat with the collision frequency of 2.0 ps^–1^. The time step used was 1 fs, and the cutoff for DFT/MM interactions was set at 15 Å. During ASM simulations, transferred hydrogen atoms were assigned a mass of 2 amu.

## 3 Results and Discussion

### 3.1 Simulations of the Michaelis Complex

In order to study the dynamic behavior of hASNaseIII, classical MD simulations were run for both the apo enzyme and the Michaelis complex. As evidenced by the RMSD, the protein and the substrate were stable during the simulation time (see Figure S4). The relative positions of the active site residues around the substrate were conserved throughout the simulations in all of the replicas and are highly consistent with the binding conformations observed for the product (PDB code 4PVS)^13^ and the acyl-enzyme (PDB code 4O0H).^13^

**Figure 2:**
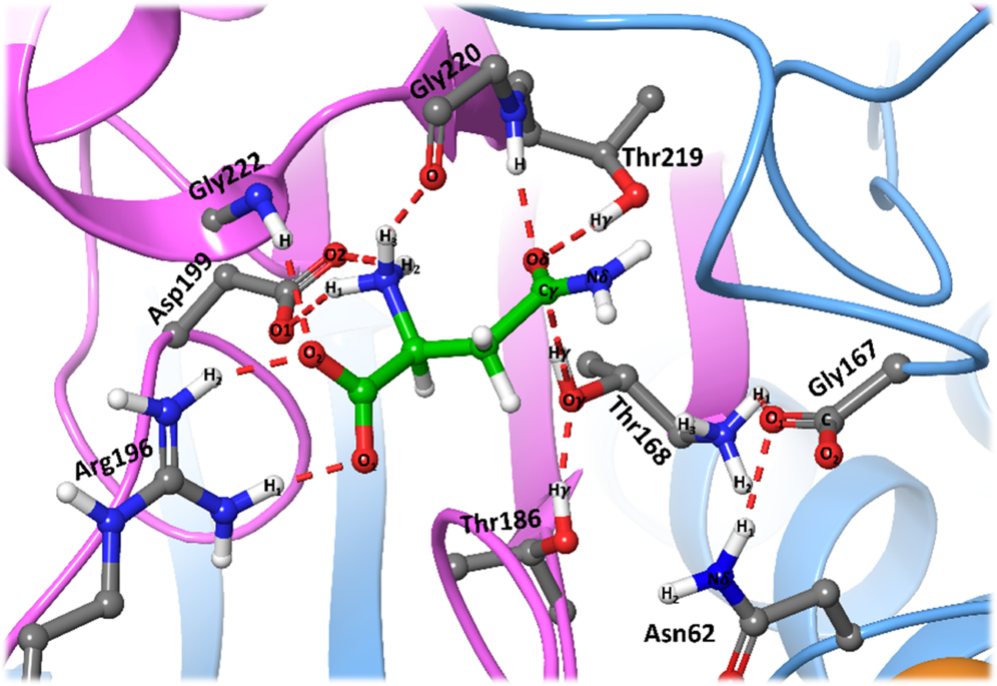
Representation of the active site of the hASNaseIII with the Asn substrate (green stick representation) as obtained from MD simulations.

Key interactions formed between the enzyme and the substrate are represented in Figure 2, while the probability distributions of these distances are given in Figure S5. The positively charged amino group (NH_3_^+^) of the substrate is stabilized by the carboxylate group of Asp199 and the carbonyl group of Gly220. The negatively charged α-carboxylic group of the substrate forms a double salt bridge with the side chain of Arg196 and a hydrogen bond interaction with the main-chain NH group of Gly222. Carbonyl oxygen of the substrate forms two hydrogen bonds with the side chain of Thr219 and the main-chain NH group of Gly220. These two residues form the so-called oxyanion hole, which can stabilize the negative charge developed on the oxygen atom of the substrate during the chemical reaction. These last interactions also assist the orientation of the NH_2_ group of the substrate toward the bulk, facilitating the elimination of the leaving group (NH_3_) during the chemical reaction. The carbonyl carbon atom (Cγ) of the substrate is found quite close to the hydroxyl oxygen of Thr168 (Oγ), with an average distance of 3.46 ± 0.44 Å. Thr168Oγ atom is the nucleophile attacking the carbonyl carbon of the substrate to form the acyl-enzyme complex. Finally, Thr186 forms a persistent hydrogen bond interaction with the hydroxyl group of Thr168, favoring its orientation for the nucleophilic attack.

**Figure 3:**
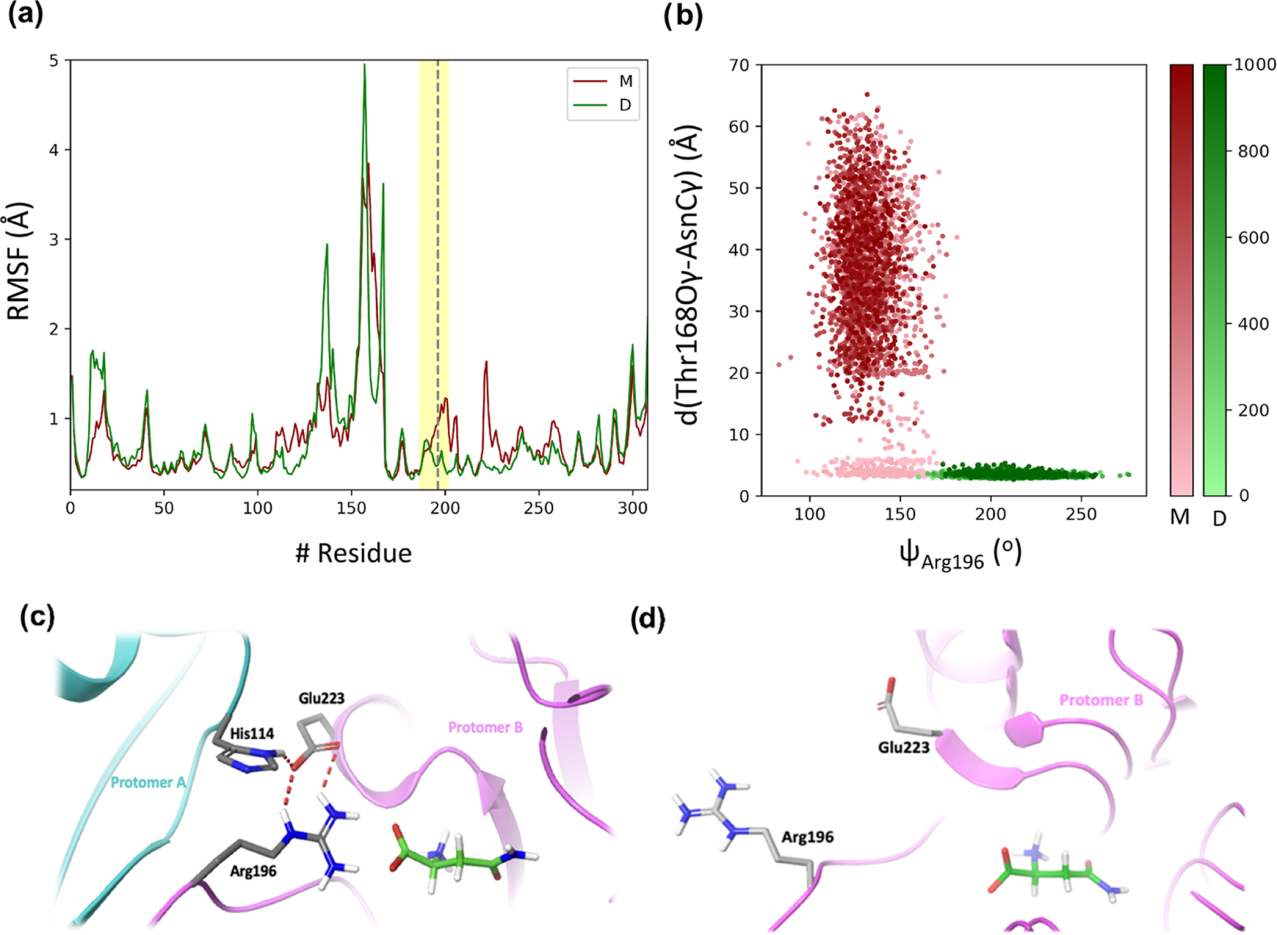
(a) RMSF of Cα of all of the residues. The red line corresponds to the monomeric (M), and the green line to the dimeric form (D) of the enzyme; the gray dashed line corresponds to the residue Arg196, while the yellow region corresponds to the whole loop. (b) Distance between Thr168Oγ and AsnCγ atoms vs ψ_Arg196_ dihedral angle in the case of monomeric (M—red) and dimeric structure (D—green). The color intensity corresponds to the time axis in ns. (c) Structure of the hASNaseIII enzyme in the ON, with the substrate in green stick representation. The lock of the ON state is due to the His114 residue of the second protomer of the dimeric structure (represented in green). (d) Structure of the OFF state of the hASNaseIII characterized by the rotation of Arg196 and Glu223 side chains.

During our simulations, sodium ions were preserved within the sodium-binding loops (residues 55–65) of both protomers in all of the replicas. The presence of sodium ions stabilizes the loops, as evidenced by the relatively low root-mean-square fluctuation (RMSF) presented in Figure 3a. Due to the network of interactions established within this loop, the side chain of Asn62, which is reported to be highly preserved within the Ntn-type enzymes,^13^ is kept oriented toward the active site (see Figure 2). Our simulations suggest that rather than behaving as a lock for Thr168, as suggested previously,^1^ Asn62 might be important in maintaining Gly167 close to the active site after the cleavage process, as evidenced by the distances between these residues shown in Figure S6. As discussed later, this Gly167 residue stabilizes the protonated amino group of Thr168 once the nucleophile is activated.

Three replicas (1 μs each) of classical MD simulations of the monomeric form of the enzyme in the Michaelis complex were performed to elucidate if this form could be catalytically active. Analysis of these simulations shows that in the case of the monomeric form, the substrate systematically leaves the active site (see Figure S7), while it always remains bound to the active site in the case of the dimeric form. This difference is due to the larger flexibility of the loop holding Arg196 and Asp199 in the case of the monomer, as evidenced by the comparison of the RMSFs (Figure 3a). Residues Arg196 and Asp199 hold the substrate in the active site through salt bridges formed between the side chains of these residues and the oppositely charged groups of the substrate (carboxyl and amino groups). Figure 3b depicts the time evolution of the ψ_Arg196_ main-chain dihedral angle and the distance between Thr168Oγ and AsnCγ in the dimer and the monomers during 1 μs of simulation. In the dimer, Arg196 is located in the interface between the two monomers, and its side chain is always oriented toward the substrate. In the monomer, the side chain of Arg196 can rotate, weakening the interaction with the substrate and allowing its departure from the active site.

The effect of the neighbor protomer on the stabilization of the Asn-bound state can be rationalized by considering a switch between two conformational states, so-called “ON” and “OFF” states.^56^ In the “ON” state, Arg196 is hydrogen-bonded to Glu223 in the position required to coordinate the substrate, while Glu223 is locked in that position by residue His114 of the second protomer (see Figure 3c). In the absence of this second protomer, Glu223 can move away from Arg196 (“OFF” state), and then this residue can change its orientation from substrate-oriented to solvent-oriented (see Figure 3d). As speculated by Loch et al.,^56^ the switch between these two states in ASNaseIII might also be triggered by a change in the protonation states of His114 and Glu223, which could explain the pH-dependent activity of this enzyme. Therefore, even though the active site residues originate from a single protomer, our results stress the importance of dimer formation for hASNaseIII activity because protomer–protomer interactions are important to keep the substrate in the active site. These results support the dimer as the only catalytically active form of hASNaseIII.

### 3.2 Protonation State of the Nucleophile

The enzymatic mechanism requires the participation of a nucleophile (Scheme 1). The analysis of the Michaelis complex shows that the hydroxyl group of Thr168 is a suitable candidate to perform this attack (Figures 2 and S5) but requires its activation by deprotonation. The N-terminal amino group of this residue could act as a base in the activation of the nucleophile, as proposed for other Ntn-hydrolases.^28,29,57,57^ To test this hypothesis, the p*K*_a_ of the terminal amino group was calculated using a thermodynamic cycle, where the enzymatic p*K*_a_ was related to the known value in aqueous solution (see Figure S1). Free energy changes were obtained using thermodynamic integration as the average of five replicas. The free energy changes obtained in the apo and holo forms of the enzyme and for free threonine in water, along with the calculated p*K*_a_ values, are given in Table S2.

The p*K*_a_ of the N-terminal group of Thr168 is calculated to be slightly smaller in the apo and holo forms (p*K*_a,apo_ = 8.6 ± 0.3 and p*K*_a,holo_ = 8.5 ± 0.3) than the reference value for free threonine in water (p*K*_a,aq_ ≈ 9.1).^58^ The important observation here is that despite the presence of the negatively charged Gly167, the p*K*_a_ of the N-terminal group of Thr168 in the enzyme does not increase with respect to the value in aqueous solution; instead, the opposite trend is observed. The origin of the p*K*_a_ shift was analyzed, quantifying the additive contributions of different groups to the electrostatic potential on the amino group; see Table S3. The effect of the negative potential created by Gly167 in the enzymatic environment, which favors the protonated form of Thr168, is compensated by the diminution in the number of water molecules around the charged amino group, as observed in the radial distribution function around the N atom in solution and in the enzyme, as shown in Figure S8. The overall effect is a less negative electrostatic potential on the N-terminal group of Thr168 in the enzyme than in solution and then a slightly lower p*K*_a_. Despite that, our results point out the protonated N-terminal group as the predominant form at pH 7.5. However, the free energy cost to deprotonate the terminal group in these conditions can be estimated to be only 1.38 ± 0.06 kcal·mol^–1^. This observation, together with the close distance between the amino and hydroxyl groups of Thr168, suggests that the former could be the base activating the nucleophilicity of the latter at a small free energy cost. Therefore, we studied the reaction mechanism considering Thr168 as unprotonated at the N-terminal group and added the free energy cost of its deprotonation to the reaction free energy profile. The determination of the reaction mechanism of the asparaginase activity was carried out in two stages: formation of the acyl-enzyme complex and deacylation by a water molecule.

### 3.3 Formation of the Acyl-Enzyme

**Figure 4:**
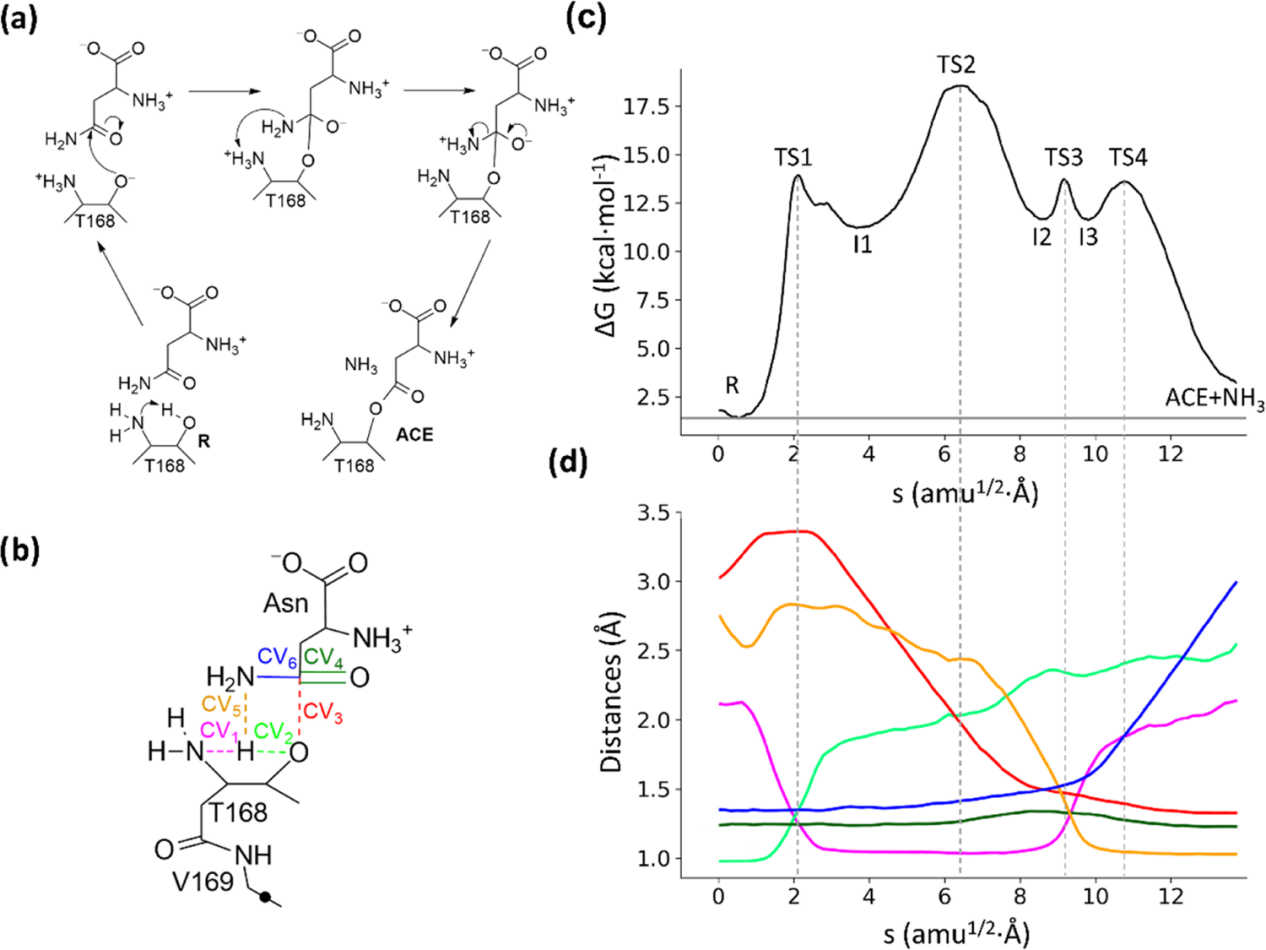
(a) Acylation mechanism (formation of acyl-enzyme, ACE) in hASNaseIII. (b) Representation of the QM region and CVs used to obtain the MFEP. The black dot represents the link atom. (c) Free energy profile along the path-CV (s) obtained at the B3LYPD3/6-31+G(d)/MM level of theory. (d) Evolution of the distances selected as CVs along the MFEP. The color code corresponds to those used in panel (b). Dashed light-gray lines indicate the position of the transition states.

We have first explored the mechanism for the formation of the acyl-enzyme complex between hASNaseIII and asparagine using free energy calculations at the DFTB3D3/MM level of theory. The most promising mechanism, in terms of the activation free energy, was then recalculated at the B3LYPD3/MM level. The resulting reaction mechanism for the formation of the covalent acyl-enzyme (ACE) involves the participation of the N-terminal group of Thr168 as the base in charge of the deprotonation of Thr168Oγ and is schematically presented in Figure 4a, together with the definition of CVs selected for its exploration (Figure 4b). We also explored the possibility of the amino group of the asparagine substrate as the base activating the nucleophile (Figure S9), but our attempts always converged to the mechanism presented in Figure 4. The free energy profile obtained by using the string method is presented in Figure 4c, together with the evolution of the CVs (Figure 4d). Note that the free energy profile shown in Figure 4c, as well as the free energies given in Table 1, already includes the free energy cost for the deprotonation of the N-terminal group of Thr168. Video S1 contains an illustrative film of the reaction mechanism.

**Table 1:** Free Energies (kcal·mol^–1^) of the Stationary Structures Corresponding to the Acyl-Enzyme Formation and Evolution of Important Distances along the MFEP (in Å)

	R	TS1	I1	TS2	I2	TS3	I3	TS4	ACE + NH_3_
free energy	1.4	14.0	11.2	18.6	11.7	13.7	11.7	13.6	2.7
CV1 (Thr168Nδ-Thr168Oγ)	2.20	1.30	1.89	2.03	2.33	2.33	2.39	2.38	2.10
CV2 (Thr168Hγ–Thr168Oγ)	0.97	1.20	1.07	1.05	1.02	1.20	1.80	2.00	2.53
CV3 (Thr168Oγ-AsnCγ)	3.07	3.36	3.00	1.86	1.43	1.47	1.46	1.36	1.30
CV4 (AsnCγ-AsnOδ)	1.22	1.25	1.23	1.30	1.36	1.32	1.32	1.27	1.23
CV5 (Thr168Hγ– AsnNδ)	2.89	2.93	2.71	2.43	2.00	1.44	1.05	1.00	1.00
CV6 (AsnCγ-AsnNδ)	1.36	1.38	1.38	1.43	1.47	1.50	1.58	1.80	3.00

**Figure 5:**
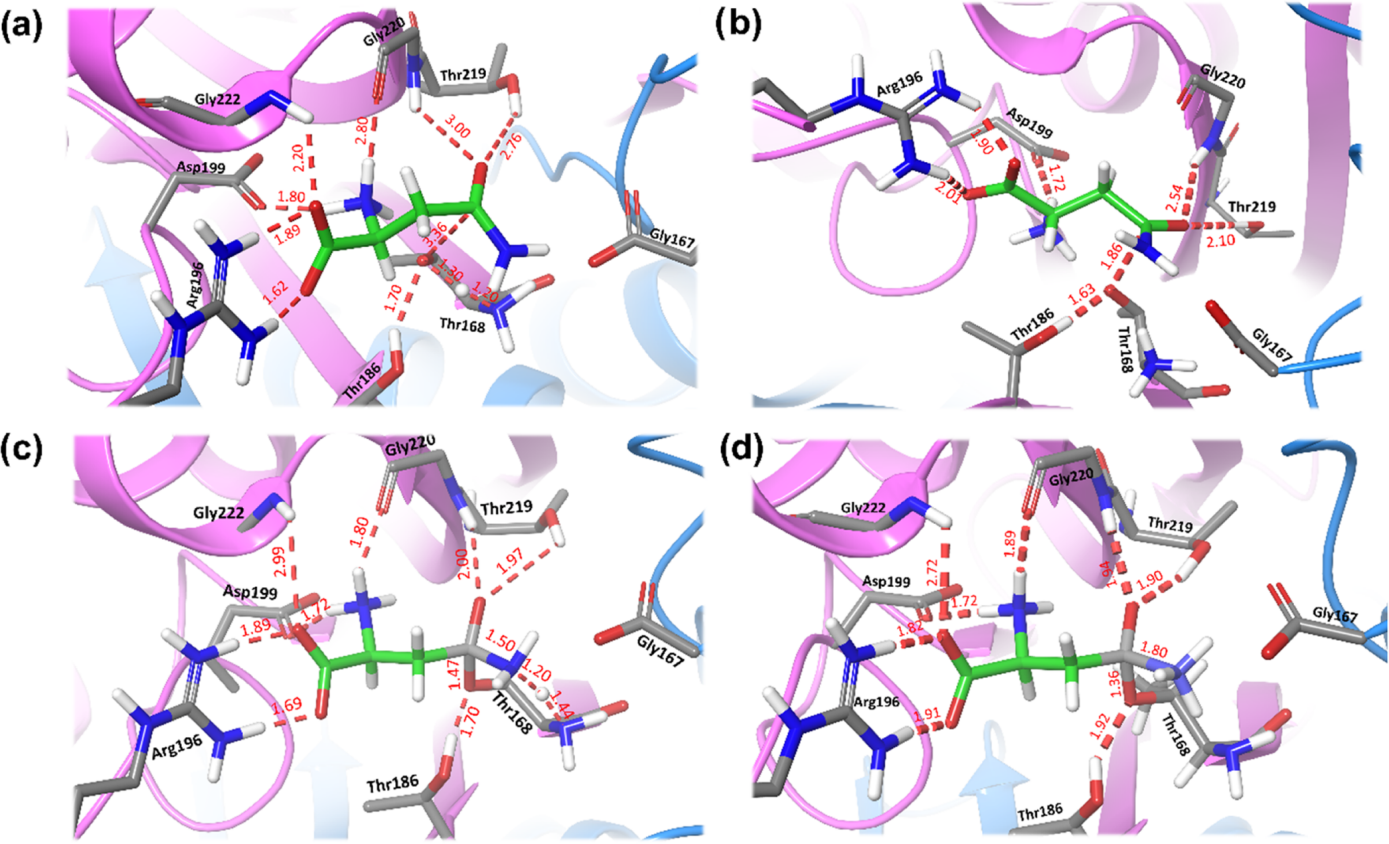
Transition states structures along with some important distances for the formation of the acyl-enzyme complex in hASNaseIII. (a) TS1, (b) TS2, (c) TS3, and (d) TS4. All distances are given in Å.

The explored mechanism displays four transition states (from TS1 to TS4), corresponding to (i) the nucleophile activation (proton transfer from Thr168Oγ to the terminal amino group); (ii) the nucleophilic attack on the Cγ atom of the substrate; (iii) proton transfer from the N-terminal group of Thr168 to the Nδ atom of the substrate, and (iv) the breaking of the Cγ–Nδ bond of the substrate and the release of ammonia. Their free energies relative to the Michaelis complex are 14.0 ± 0.4, 18.6 ± 0.7, 13.7 ± 0.9, and 13.6 ± 0.9 kcal·mol^–1^, respectively, so TS2 is the rate-limiting TS during the acylation stage. Geometries of the transition states are shown in Figure 5.

According to this mechanism, the acylation stage starts with the activation of the nucleophile by means of a proton transfer from the hydroxyl oxygen to the amino group of Thr168 (see the evolution of CV1 and CV2 in Figure 4d). This process is assisted by the hydroxyl group of Thr186 that stabilizes the negative oxygen of the nucleophile (see Figure 5a), and the hydrogen bond distance between the hydroxyl groups of Thr186 and Thr168 diminishes from 2.19 Å in the reactant state to 1.70 Å in TS1. Thr186 maintains a strong hydrogen bond interaction with the Thr168Oγ atom during the rest of the acylation process, as can be observed in Figure 5. The stabilizing role of Thr186 agrees with the observation that the T186 V lacks asparaginase activity.^59^

The second transition state (TS2) presents a higher free energy for the proposed mechanism and corresponds to the nucleophilic attack of Thr168 on the Cγ atom of the substrate. The Thr168Oγ-AsnCγ distance describing this process (CV3) is reduced up to 1.76 Å at TS2 (see Figure 5b). Simultaneously, the length of the carbonyl bond (CV4) increases from a double bond to a single bond. This TS2 is stabilized by the oxyanion hole residues (Thr219 and Gly220) that provide hydrogen bonds to the carbonyl oxygen of the substrate, where a negative charge is built up. The hydrogen bond distance between the carbonyl oxygen and the hydroxyl group of Thr219 is decreased from 2.76 Å in the reactants complex to 2.10 Å in TS2, while the distance to the main-chain NH group of Gly220 diminishes from 3.00 to 2.54 Å. Interestingly, the T219V and T219A mutants do not present asparaginase activity,^59^ presumably due to the reduced ability of the enzyme to stabilize TS2 through hydrogen bond interactions.

Third and fourth transition states, TS3 and TS4, correspond to proton transfer from the amino group of the nucleophile to the amino group of the substrate and the release of ammonia after breaking the Cγ–Nδ bond, respectively. This is reflected in the evolution of CV1, CV5, and CV6 in Figure 4d. CV1 and CV5 describe the proton transfer from the amino group of Thr168 to that of the substrate. At TS3, the proton is almost halfway between the donor and acceptor nitrogen atoms, with the distances being 1.32 and 1.20 Å, respectively (see Figure 5c). The breaking of the Cγ–Nδ bond is described by CV6 that reaches a value of 1.58 Å after the proton transfer and 1.80 Å at TS4, before it becomes completely broken at ACE (Figure S10). Simultaneously to the release of ammonia, the length of Cγ–Oδ bond is shortened to the value corresponding to a double bond, as given by the evolution of CV4. After breaking the Cγ–Nδ bond, the free energy monotonically drops to the product state, which presents a free energy of 2.7 ± 0.5 kcal·mol^–1^ with respect to the Michaelis complex.

In order to complete the enzymatic cycle, we also evaluated the free energy change corresponding to the release of ammonia from the active site to the bulk solution. This process was computed using the thermodynamic cycle represented in Figure S2, where an ammonia molecule disappears from the active site and appears in the bulk (see the SI for details). The total Gibbs free energy obtained for the NH_3_ elimination from the active site was −4.47 ± 0.04, kcal·mol^–1^. This value is comparable with the values found for the release of ammonia in other enzymatic systems,^60^ and can be explained by the additional hydrogen bond that NH_3_ can form in bulk water in comparison to the active site. Addition of the free energy associated with ammonia release results in a reaction free energy of −1.8 ± 0.6 kcal·mol^–1^ for the acylation stage relative to the Michaelis complex.

### 3.4 Hydrolysis of the Acyl-Enzyme and Enzyme Regeneration

**Figure 6:**
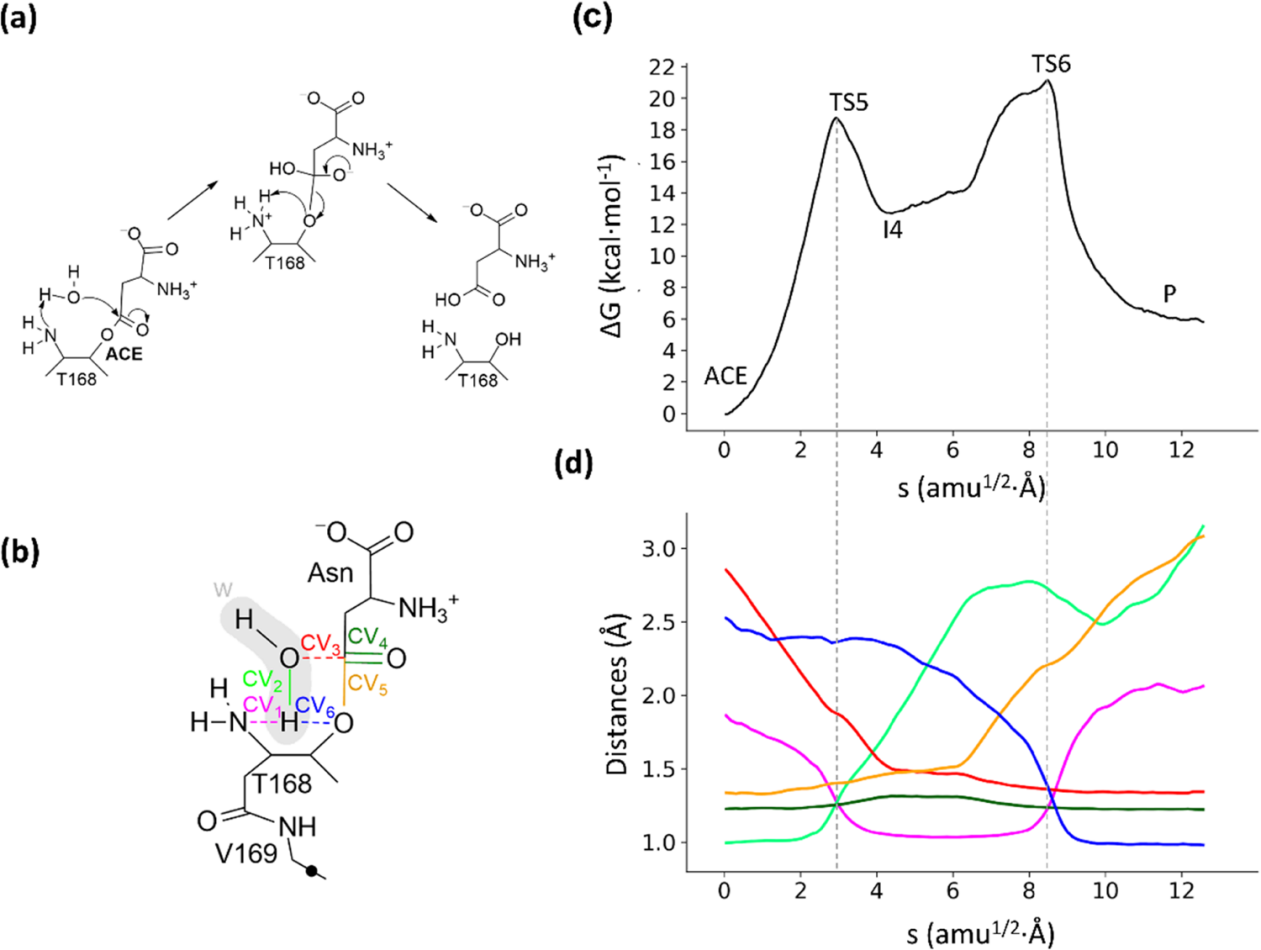
(a) Acyl-enzyme hydrolysis reaction mechanisms in hASNaseIII. (b) Representation of the QM region and CVs used to obtain the MFEP. The black dot represents the link atom. (c) Free energy profile along the path-CV (s) obtained at the B3LYPD3/6-31+G(d)/MM level of theory. (d) Evolution of the distances selected as CVs along the MFEP. The color code corresponds to that represented in panel (b). Dotted light-gray lines indicate the position of the transition states.

**Figure 7:**
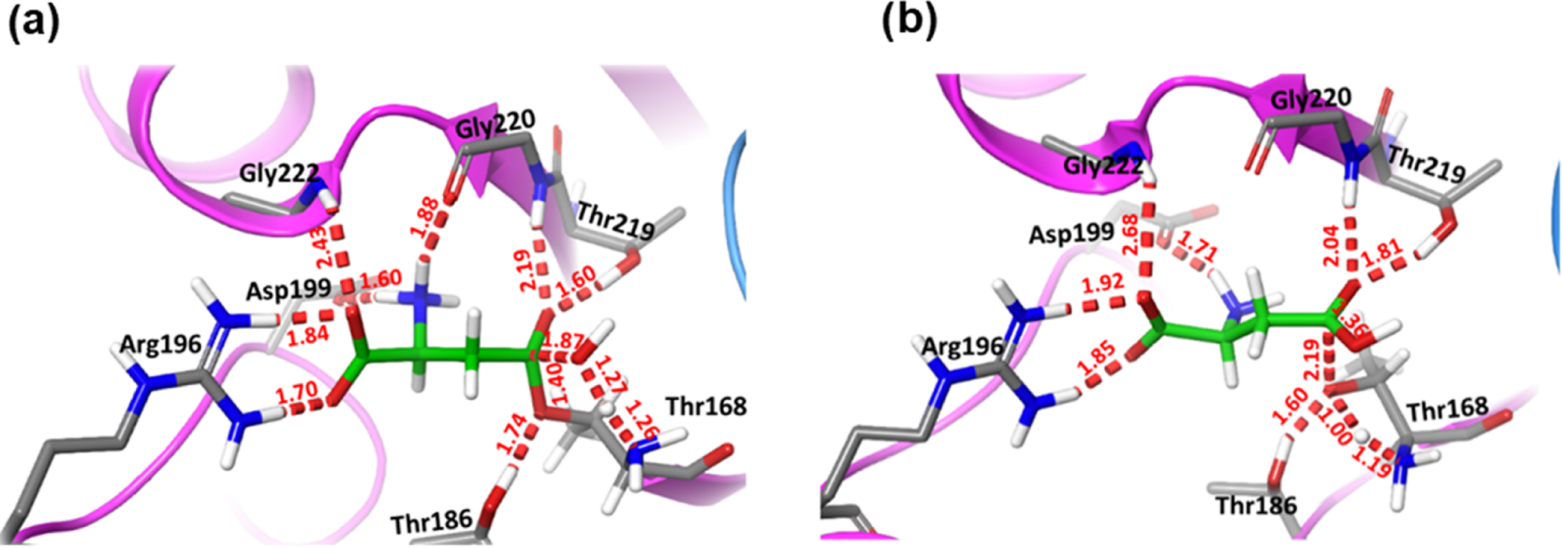
Transition-state structures along with some important distances. On the left TS5 and TS6 on the right. All distances are given in Å.

The second part of the enzymatic cycle consists of the hydrolysis of the previously formed acyl-enzyme (ACE) by a water molecule. To prepare the initial structures for the string method, the acyl-enzyme structure obtained in the previous calculation was relaxed during 1 μs of the classical MD simulation. The parameters for the acyl-enzyme, with a covalent bond between the substrate moiety and Thr168, were obtained, as explained in the Methodology section. We then explored different mechanistic possibilities for the hydrolysis of the acyl-enzyme complex using free energy calculations at different levels of theory (see Figure S11). The explored mechanisms involved the participation of one or two water molecules and different active site residues. The computed mechanism with the lowest activation free energy was then relocalized at the B3LYPD3/MM level. This mechanism is schematically presented in Figure 6a, while the QM region and the set of CVs employed are presented in Figure 6b. The free energy profile obtained with the string method is presented in Figure 6c, together with the evolution of the corresponding CVs (Figure 6d). Free energy values of the stationary points of the PMF in Figure 6c, together with the values of corresponding CVs, are provided in Table 2, while the transition states found along the path are represented in Figure 7. Video S2 illustrates the hydrolysis mechanism.

**Table 2:** Free Energies (in kcal·mol^–1^) of the Stationary Structures Corresponding to Hydrolysis Stage and Evolution of Important Distances along the MFEP (in Å)

	ACE	TS5	I4	TS6	P
free energy	0.0	18.6	12.7	20.9	5.8
CV1 (Thr168Nδ-Hw)	1.87	1.26	1.05	1.19	2.02
CV2 (Hw-Ow)	0.99	1.27	1.81	2.74	2.90
CV3 (Ow-AsnCγ)	2.85	1.87	1.50	1.36	1.33
CV4 (AsnCγ-AsnOδ)	1.23	1.25	1.30	1.25	1.22
CV5 (AsnCγ-Thr168Oγ)	1.34	1.40	1.46	2.19	2.99
CV6 (Thr168Oγ-Hw)	2.53	2.36	2.33	1.41	1.00

The deacylation stage starts with the deprotonation of the hydrolytic water molecule by the basic amino group of Thr168, followed by the nucleophilic attack on the Cγ atom of the substrate by the water oxygen atom (Ow). These two processes occur in a rather concerted process, as evidenced by the evolution of the Thr168Nδ-Hw, Hw-Ow, and Ow-AsnCγ distances (CV1, CV2, and CV3 in Figure 6d). The corresponding TS (TS5) presents a free energy of 18.6 ± 0.7 kcal·mol^–1^ relative to that of the acyl-enzyme complex. TS5 is stabilized again by the oxyanion hole residues since, upon the nucleophilic attack, a negative charge builds up again on the carbonyl oxygen atom of the substrate (Figure 7a).

After nucleophilic attack, a tetrahedral intermediate (I4) is formed. From this intermediate, the reaction proceeds with the proton transfer from the positively charged amino group of Thr168 to the acyl oxygen, as shown by the evolution of the Thr168Nδ-Hw and Thr168Oγ-Hw distances (CV1 and CV6 in Figure 6d) and the breaking of the acyl-enzyme bond, reflected in the increase of the AsnCγ-Thr168Oγ distance (CV5). This last step is found to be the rate-determining step of the global process since the free energy barrier relative to the acyl-enzyme complex is 20.9 ± 1.2 kcal·mol^–1^. TS6 is also stabilized by the oxyanion hole residues (Thr219 and Gly220), as seen in Figure 7b, and by a strong hydrogen bond established between the hydroxyl group of Thr186 and the acyl-oxygen atom. The product obtained after TS6 is an aspartic acid protonated in its side-chain group.

To complete the enzymatic cycle, one additional string calculation was performed to explore the regeneration of the protonation state of the enzyme by means of the transfer of a proton from the carboxylic group of the newly formed aspartic acid to the amino group of Thr168. The set of CVs used to model this stage, their evolution along the MFEP, the free energy profile, and a representation of the TS structure are given in the SI section (Figure S12 and Table S5). The structure of the corresponding transition state, along with the important distances, is given in Figure S13. A low free energy barrier was obtained for this proton transfer process, 5.2 ± 0.8 kcal·mol^–1^, followed by a monotonic drop in the free energy, resulting in overall exergonic enzymatic process with a reaction free energy equal to −7.2 ± 0.6 kcal·mol^–1^ with respect to the Michaelis complex.

### 3.5 Complete Reaction Cycle in hASNaseIII

**Figure 8:**
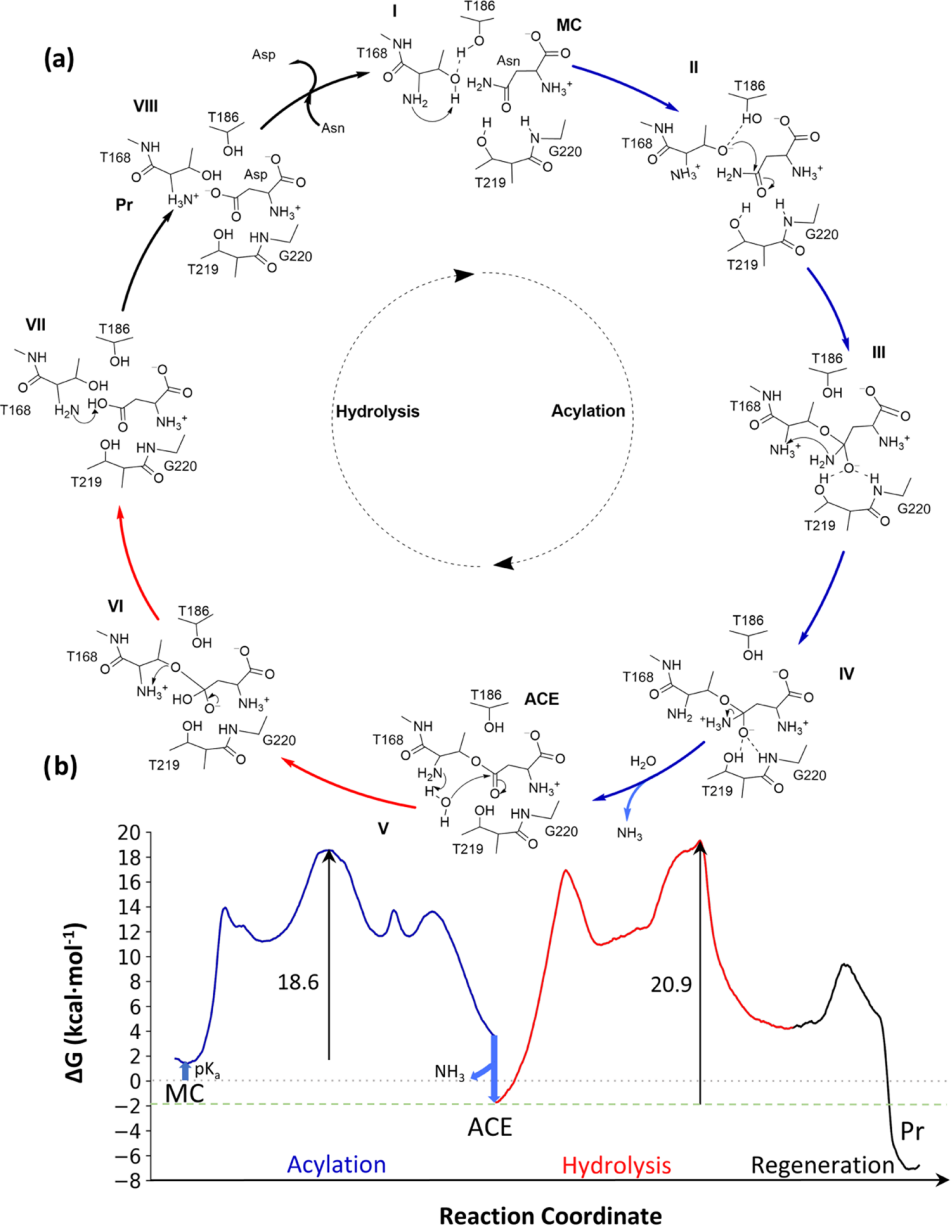
Hydrolysis mechanism of asparagine in hASNaseIII as deduced from our simulations. (a) Schematic representation of the reaction mechanism. (b) Free energy profile associated with the reaction mechanism. The acylation stage is displayed in blue, the deacylation stage in red, and the regeneration stage in black.

The complete catalytic cycle of hASNaseIII and its associated B3LYPD3/6-31+G(d)/MM free energy profile are listed in Figure 8. The final free energy profile was obtained by joining all three free energy profiles discussed above, along with the energy changes due to the change in the protonation state of the nucleophile and the departure of ammonia from the active site.

As seen in Figure 8b, the rate-limiting step is associated with the breaking of the acyl-enzyme bond, reaching a free energy barrier of 20.9 kcal·mol^–1^. This value is in good agreement with the value derived from the experimental rate constant,^59^ 17.5 kcal·mol^–1^. One should consider that zero-point energy corrections can be expected to lower the activation free energy due to the participation of a proton transfer, which could improve the agreement between the theoretical and experimental values. As said before, the overall cycle was found to be exergonic, with a reaction free energy of −7.2 kcal·mol^–1^.

For further confirmation of the mechanism, the structure of the covalent acyl-enzyme complex was compared with the corresponding X-ray structure (4O0H). The resulting overlap is shown in Figure S14. The value of the RMSD after the alignment between the two structures was found to be only 0.97 Å. The free energy profile given in Figure 8b also explains the possibility of crystallizing this covalent intermediate. Note that the reaction step for the formation of the acyl-enzyme is irreversible because of the release of ammonia to the bulk and that the deacylation is the rate-limiting step, resulting in a relatively long-lived intermediate.

## 4 Conclusions

In this work, the mechanism for the hydrolysis of l-asparagine catalyzed by human ASNase type III has been studied by means of classical and hybrid QM/MM simulations. A computational model was prepared by modification of the X-ray structure of the acyl-enzyme intermediate (40OH), where missing residues were modeled using AlfaFold2. Classical MD simulations revealed key interactions established between the substrate and active site residues in the Michaelis complex. Even though the active site residues stabilizing the substrate originate from a single protomer, our simulations revealed the importance of the dimeric form of the enzyme in stabilizing the substrate in the active site. Protomer–protomer interactions are important to keep the side chain of Arg196 in contact with the substrate and to stabilize the bound state. We also run alchemical free energy calculations, coupled with thermodynamic integration, to investigate the p*K*_a_ of the N-terminal group of Thr168, which seems to be the best candidate to act as the base responsible for the activation of the nucleophile, the hydroxyl group of the same residue. Results of these free energy calculations revealed that the unprotonated Thr168 can be reached at a relatively small free energy cost under experimental conditions (pH 7.5 and T = 310 K). The p*K*_a_ value of the N-terminal group was found to be slightly lower in the enzyme (8.6 and 8.5 for the apo and holo forms, respectively) than in aqueous solution (9.1).

The reaction mechanism was explored by starting from a deprotonated N-terminal group in Thr168. This group can then act as the base in charge of proton abstraction from the hydroxyl group of the same residue. The complete catalytic cycle of the studied mechanism displayed seven steps (see Figure 8): (i) the activation process of the nucleophile, (ii) the nucleophilic attack to form the covalent acyl-enzyme complex, (iii) the proton transfer to the leaving amino group, (iv) the release of ammonia from the substrate to the bulk, (v) activation of a water molecule and its nucleophilic attack on the acyl-enzyme complex, (vi) acyl-enzyme bond breaking, and (vii) regeneration of the protonation state of the enzyme. The rate-determining step corresponds to the second step of the deacylation stage, and the value obtained for the free energy barrier is 20.9 kcal·mol^–1^, in good agreement with the experimentally derived value, 17.5 kcal·mol^–1^. Analysis of the structures of the transition states revealed the importance of residues Thr186 and Thr219 in their stabilization, which explains the lack of enzymatic activity in Thr219Val, Thr219Ala, and Thr186Val mutants. The overall process is exergonic, with a reaction free energy of −7.2 kcal·mol^–1^. Moreover, the overall free energy profile of the whole cycle shows that the acyl-enzyme complex is a stable intermediate formed after an irreversible step, which explains why this complex can be observed by X-ray techniques. The reaction mechanism presented here can be also probably operative for the whole family of Ntn-hydrolase enzymes. Finally, the findings of the present work could also be useful to assist in the design of new enzymes to be used as treatment of acute lymphoblastic leukemia.

## Data Availability

https://github.com/emedio/hASNaseIII/ The github repository contains all of the input files used in MD simulations, including QM/MM ASM simulations. It also includes parameter files (prmtop) of the dimer form of the protein with protonated N-terminal and unprotonated N-terminal. PDB structures of all of the transition states along the thermodynamic cycle are provided as well. Repository also contains video animations of the acylation and hydrolysis step of the thermodynamic cycle.

## Supporting Information Available

The Supporting Information is available free of charge on the ACS Publications Web site. Detailed technical information about MD simulations; iInformation about thermodynamic integration calculations; figures describing thermodynamic cycles followed in the free energy calculations; tables containing details about free energy calculations; a table with the contributions of different functional groups to the electrostatic potential on the hydrogen atoms of protonated amino group of Thr168; a figure depicting the impact of QM level on the acylation free energy profile; RMSD plots of MD simulations of three replicas; distributions plots of the important distances within the active site of the dimeric protein; probability density plot of the distance between the Asn62Nδ and the Gly167C; time evolution figures of the Thr168Oγ-AsnCγ(Asn) distance of the monomer form of the enzyme; plot of the radial distribution functions of the water environment of N-terminal threonine and free threonine in solution; acylation mechanism, with the substrate amino group acting as the base in charge of the activation of the nucleophile and evolution of the CVs during convergence of the string; a figure representing the structure of the product of the acylation step; alternative mechanisms for the hydrolysis step; a figure depicting the reaction mechanism for the regeneration part of the catalytic cycle; a table of the free energy of the stationary structures within the regeneration mechanism; representation of the TS structure of the regeneration step of the catalytic cycle; overlap between theoretically obtained acyl-enzyme structure and the X-ray structure of the acyl-enzyme; videos of the reaction mechanisms (PDF)
Illustrative film of the reaction mechanism (Video 1) (MP4)
Illustration of the hydrolysis mechanism (Video 2) (MP4)
