# Supplementary material for: Elucidation of the Active Form and Reaction Mechanism in Human Asparaginase Type III Using Multiscale Simulations
Source: J Chem Inf Model. 2023 Aug 28;63(17):5676–88. doi: 10.1021/acs.jcim.3c00900 (PMC10852353; doi:10.1021/acs.jcim.3c00900)
Supplement: Supplementary file 1 — ci3c00900_si_001.pdf [file ci3c00900_si_001.pdf]

Supporting Information

Elucidation of the Active Form and Reaction Mechanism in Human Asparaginase Type III Using Multiscale Simulations

Milorad Andjelkovic[1], Kirill Zinovjev[1], Carlos Alberto Ramos-Guzmán[1,2], Jose Javier Ruiz- Pernía*[1], Iñaki Tuñón*[1]

[1] Departamento de Química Física, Universidad de Valencia, 46100 Burjassot (Spain)
[2] Instituto de Materiales Avanzados, Universidad Jaume I, 12071 Castelló (Spain)

*To whom correspondence should be addressed
ignacio.tunon@uv.es
j.javier.ruiz@uv.es

MD equilibration. Minimization was carried out in several cycles, each consisting of 50000 steps, out of which first 10000 steps with steepest descent method and then switched to conjugate gradient method, until a root-mean-squared gradient of around 10^{-4} kcal·mol^{-1} Å was reached. Minimized structures were then heated to 310 K using Langevin dynamics with a collision frequency equal to 5.0 ps^{-1} and a linear heating ramp, rising the temperature from 0 to 310 K. During the heating simulations, periodic boundary conditions were applied with isotropic position scaling and the time step was f 1 fs. During heating, a mild restraint parabolic potential was applied to protein backbone atoms with a force constant of 20 kcal·mol^{-1}·Å^{-2}. If the system included substrate, parabolic restrains between the substrate and active site residues distances were also applied. Heated structures were then equilibrated while slowly releasing applied restrains (~ 1 kcal·mol^{-1}·Å^{-2}·ns^{-1}). The time step was increased to 2 fs since SHAKE[1] algorithm was applied to constraint bonds involving hydrogen atoms. After releasing all restrains, equilibrated structures entered a production stage of 1000 ns simulations in the NVT ensemble performed in Amber18 GPU version of pmemd,[2,3] keeping all the simulation parameters the same as during the equilibration period. In order to achieve better sampling, three replicas of 1 µs were run on each system.

Thermodynamic Integration Calculations. The pK_a of the N-terminal group of Thr168 was calculated using the thermodynamic cycle presented in Figure S3. Equation (S1) connects the pK_a shift and the change in the interaction free energy of the N-terminal group in the protonated and unprotonated forms:

$$pK_{prot} = pK_{aq} + \frac{1}{2.303\,kT}\Delta\Delta G \qquad \text{(S1)}$$

$\Delta\Delta G$ represents the difference in free energy change obtained when deprotonating the amino group of a threonine in the protein environment and in aqueous solution, $\Delta\Delta G = \Delta G_{B-D} - \Delta G_{A-C}$, k is Boltzmann constant, T is the temperature and pK_{prot} and pK_{aq} stand for the pK values of the terminal group in protein environment and in aqueous solution, respectively. These free energy changes were evaluated from the free energy differences resulting of the alchemical transformations of the neutral into the protonated residue in aqueous solution and in the protein environment (see Figure S2):

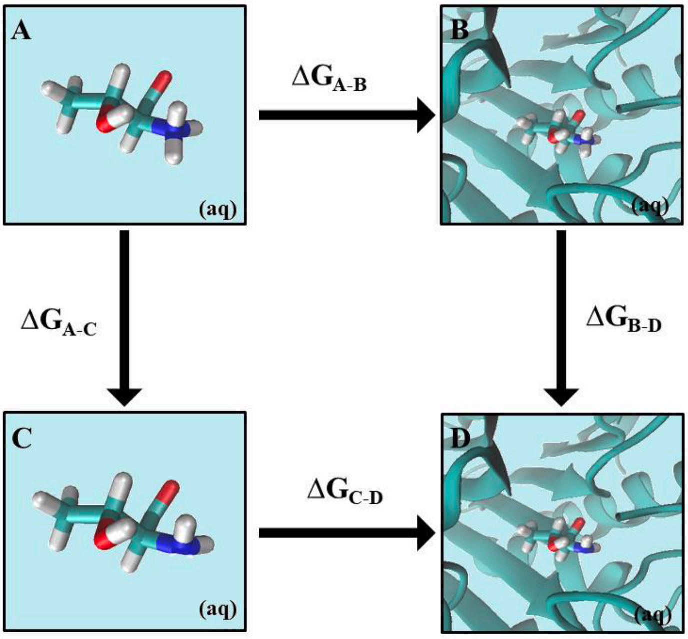

Figure S1. Thermodynamic cycle used for the evaluation of the change in the protonation state of the amino group of threonine in water and in the protein. The blue background represents water environment. States A and C correspond to protonated and deprotonated threonine in water, respectively. States B and D correspond to protonated and deprotonated threonine being part of hASNaseIII enzyme, respectively. Alchemical transformations were performed along vertical axes A→C and B→D.

The thermodynamic Integration (TI) method was employed as a free energy estimator. The free energy was calculated by numerical integration of the derivative of free energy over a coupling parameter (λ), driving the alchemical transformation from an unprotonated to a protonated N-terminal group, as given by equation (S2):

$$\Delta G = \int_0^1 \langle \frac{\partial U(\lambda)}{\partial \lambda} \rangle_\lambda \, d\lambda \qquad \text{(S2)}$$

Free energy calculations were performed in Amber18[4] using dual topology approach and following a recently published protocol for GPU-based simulations.[5] Briefly, the temperature was kept at 310 K using Langevin dynamics with the collision frequency was 5.0 ps^{-1}. The pressure was kept constant at 1.01325 bar with Monte Carlo barostat and a pressure relaxation time of 2.0 ps. The unique atom that is disappearing along the alchemical transformation (the additional proton) was set in the soft-core region for both van der Waals and electrostatic interactions. All simulations were run in pmemd.cuda in GPU, and the time step was set equal to 1 fs. In each of the five replicas used to obtain a reliable averaged estimation (Table S1), alchemical transformation was done using 9 different λ values, following Gaussian quadrature method (λ= 0.01592, 0.08198, 0.19331, 0.33787, 0.5, 0.66213, 0.80669, 0.91802, 0.98408). The five separate replicas were run for 5 ns long for each λ value. The first 1 ns of each simulation was considered as an equilibration step at the corresponding λ window and therefore was skipped for post-analysis of dU/dλ. Finally, five free energy changes derived from the corresponding replicas

were averaged arithmetically. This average value of the free energy change, corresponding to the alchemical transformation of the residue in water, was then subtracted from the identically obtained and averaged in protein environment, and this difference (ΔΔG) was used in eq (S1). The error was calculated as the standard error of the mean (SEM), dividing the standard deviation (STD) by the square root of the number of replicas.

Table S1. Results of five replicas performed to calculate the free energy change (in kcal·mol^{-1}) associated to the alchemical transformations between the unprotonated and protonated forms of threonine performed in aqueous, apo protein and holo protein environments. Average values (mean) are given with the corresponding standard deviations (std).

environment	rep	ΔG_{A-C}	environment	rep	ΔG_{B-D}	Environment	rep	ΔG_{B-D}
	1	-166.34		1	-166.05		1	-166.71
	2	-166.42	apo	2	-167.02	holo	2	-165.90
aqueous	3	-166.58	protein	3	-165.00	protein	3	-165.34
	4	-166.51		4	-165.58		4	-164.70
	5	-166.42		5	-165.12		5	-165.14
mean		-166.45	mean		-165.75	Mean		-165.56
std		0.18	std		1.41	Std		1.39

Table S2. Free energies for the alchemical transformation of threonine protonation state in the apo and holo forms with respect to the water. pK_a shifts and pK_a values of the threonine residue in the apo and holo form. Free energy cost associated to the deprotonation of the threonine residue in apo and holo protein at the pH=7.5 and T=310 K, and calculated probabilities of the protonated and deprotonated form of threonine residue at pH=7.5 and T=310 K.

Form	apo	holo
ΔG_{B-D} (kcal·mol^{-1})	-165.75 $\pm$ 0.63	-165.56 $\pm$ 0.62
$\Delta\Delta G = \Delta G_{B-D} - \Delta G_{A-C}$ (kcal·mol^{-1})	-0.70 $\pm$ 0.45	-0.89 $\pm$ 0.48
$pK_{prot} - pK_{aq}$	-0.48 $\pm$ 0.33	-0.62 $\pm$ 0.34
pK_{prot}	8.62 $\pm$ 0.33	8.47 $\pm$ 0.34
$\Delta G(deprotonation)_{pH=7.5,T=310\ K}$ (kcal·mol^{-1})	1.58 $\pm$ 0.06	1.38 $\pm$ 0.06
$P(protonated)_{pH=7.5,T=310\ K}$	0.92	0.87
$P(deprotonated)_{pH=7.5,T=310\ K}$	0.08	0.13

Table S3. Contributions of different groups to the electrostatic potential (J·C^{-1}) on the hydrogen atoms of protonated amino group of Thr168 in the Michaelis complex and in aqueous solution.

Group	Michaelis complex	Aqueous solution
Total potential	-5.60 ± 0.02	-5.95 ± 0.02
Waters	-0.68 ± 0.04	-5.95 ± 0.02
Gly167 COO^-	-1.48 ± 0.05	
Substrate NH_3^+	+0.48 ± 0.04	
Substrate COO^-	-0.53 ± 0.04	

The free energy change corresponding to NH_3 leaving the active site (-ΔG_{bind}) can be obtained by combining different terms that appears in the thermodynamic cycle of figure S2:

$$-\Delta G_{bind} = \Delta G^{*}_{bulk} + \Delta G^{V^o \rightarrow rest}_{decoupled} - \Delta G^{*}_{site} \quad \text{(S3)}$$

The first (ΔG^{*}_{bulk}) and last (ΔG^{*}_{site}) terms correspond to the free energies of the appearance of NH_3 in the water and decoupling NH_3 in the bound state, respectively. Both terms were calculated as an average of five separate replicas (Table S2). The softcore potential was applied to the whole NH_3, therefore allowing SHAKE to be used. Replica exchange was applied to ensure better sampling and each window simulation was run for 20 ns. In order to keep the volume constant to enable replica exchange between different λ windows production simulations were run in the NVT ensemble. In order to maintain NH_3 in the active site during all the stages of the alchemical transformation a simple restraining potential has been applied to the distance between N of NH_3 and Cγ of substrate. The free energy associated with restraining the decoupled ammonia ($\Delta G^{V^o \rightarrow rest}_{decoupled}$) has been calculated as:

$$\Delta G^{V^o \rightarrow rest}_{decoupled} = -RTln\left(\frac{Q}{V^o}\right) \quad \text{(S4)}$$

where Q is given as:

$$Q = \int_0^{\infty} 4\pi r^2\, e^{-\beta U_{rest}(r)} dr \quad \text{(S5)}$$

The force constant of the applied semi-parabolical restraining potential of the form $U_{rest} = k(r - r_0)^2$ was *50* kcal·mol^{-1}·Å^{-2}, where r stands for the AsnCγ-AsnNδ distance. The limiting distance of the restraining potential (r_0) was set to 3.5 Å which corresponds to the distance measured in the last node of the string calculation.

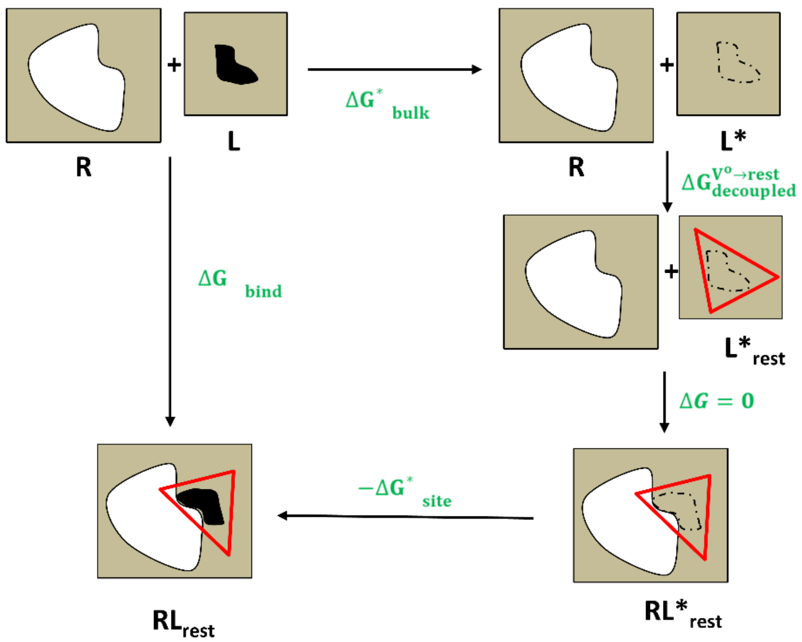

Figure S2. Thermodynamic cycle used to evaluate free energy change of NH_3 leaving the active site. R: unbound protein. L: unbound NH_3 in water. L*: decoupled NH3. RL: protein-NH_3 complex.

During the evaluation of a standard binding free energy, equation (S3) should also include additional free energy correction due to the release of the restrains on the coupled NH_3 in the active site ($\Delta G_{coupled}^{rest \to site}$)[6,7]. However, since the free energy changes we are calculating correspond to the release of a restrained NH_3 (as obtained from the string calculation), then this term is omitted. Taking all into account, calculated correction is 2.74 kcal·mol^{-1}. The term ΔG_{bulk}^{*} is calculated to be 4.02 ± 0.03 kcal·mol^{-1}, while ΔG_{site}^{*} is -2.29 ± 0.03 kcal·mol^{-1}. Therefore, the total free energy of NH_3 leaving the active site was found to be -4.47 ± 0.04 kcal·mol^{-1}.

Table S4. Free energy changes associated to alchemical transformations performed in water and protein environments for the release of ammonia from the active site of hASNaseIII to the bulk. Free energy values (in kcal·mol^{-1}) were estimated using TI and each average value is given with the corresponding standard deviation.

Environment	replica	ΔG_{bulk}^{*}	environment	replica	ΔG_{site}^{*}
	1	-4.00		1	-2.27
	2	-4.02		2	-2.25
aqueous	3	-4.05	protein	3	-2.32
	4	-4.00		4	-2.31
	5	-4.03		5	-2.28
mean		4.02	mean		-2.29
std		0.07	std		0.07

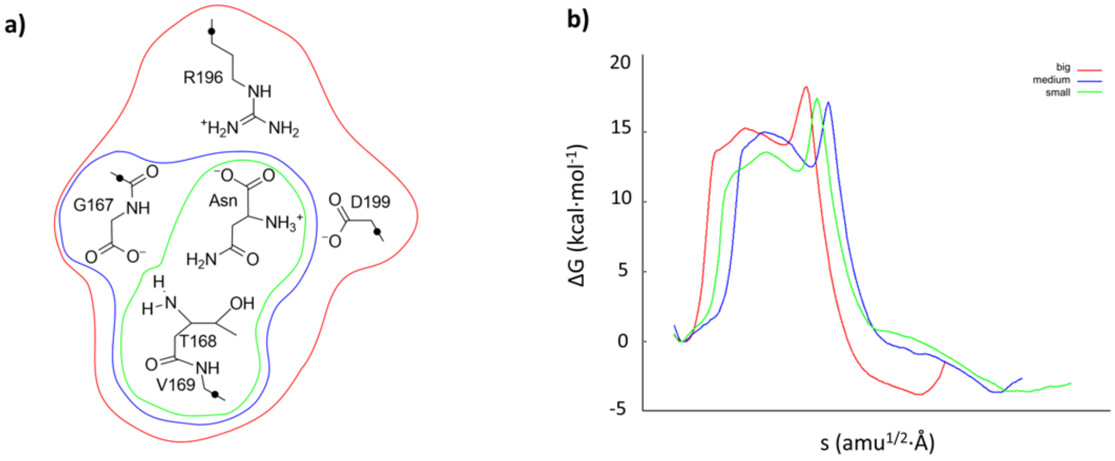

Figure S3. Effect of the size of the QM region (a) on the free energy profile corresponding to the acylation mechanism (b). Each QM region is represented with one color.

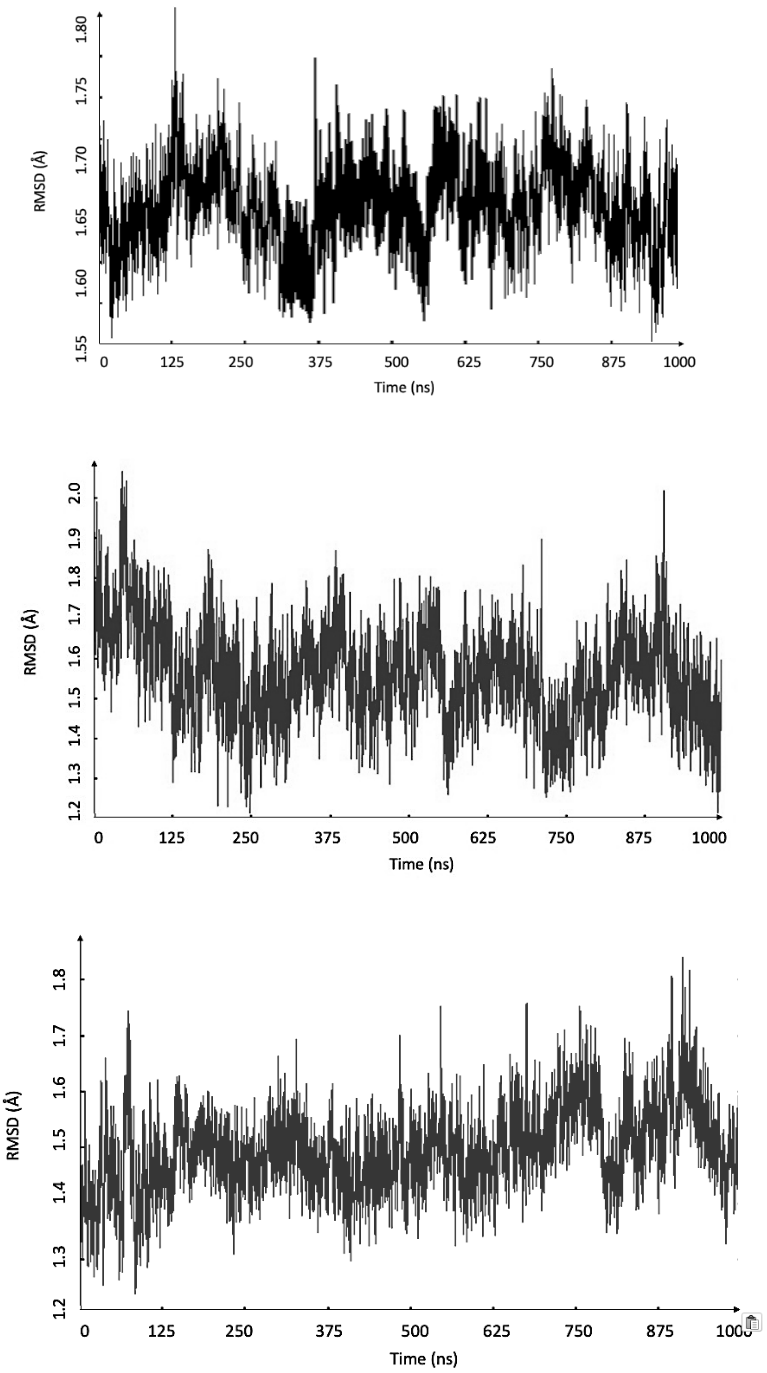

Figure S4. Root mean square deviation along the MD simulations (three replicas) of the Michaelis complex of dimeric form of hASNaseIII with substrate in the active site.

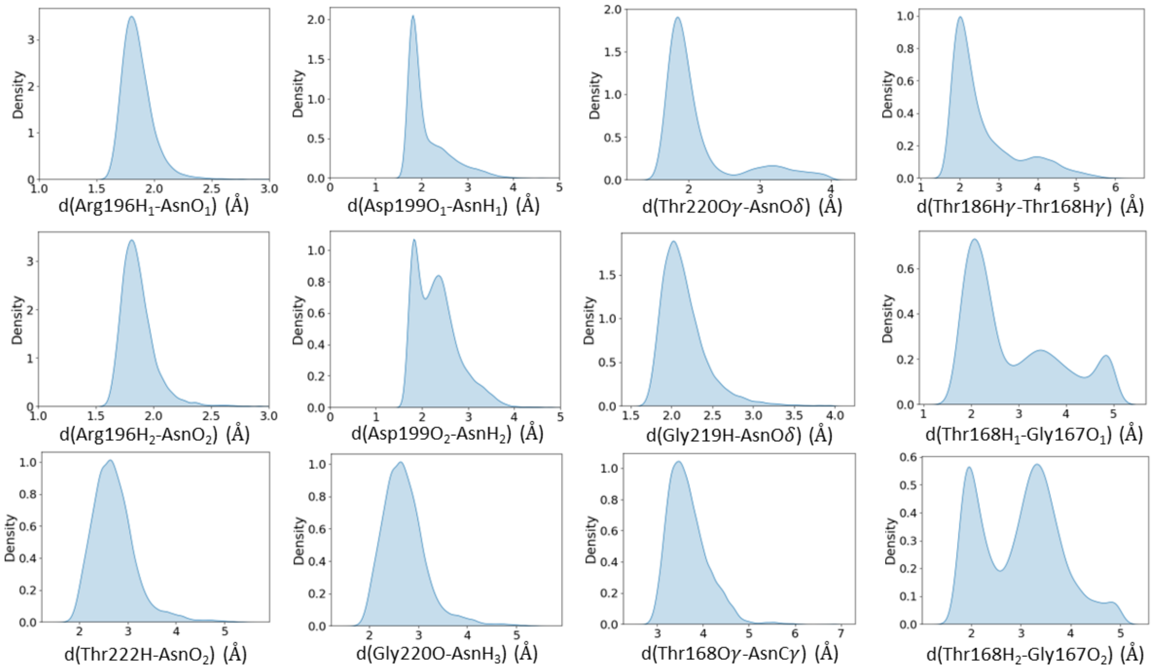

Figure S5. Distributions of the important distances in (Å) obtained over three replicas of 1 μs of classical molecular dynamic simulation run on the Michaelis complex.

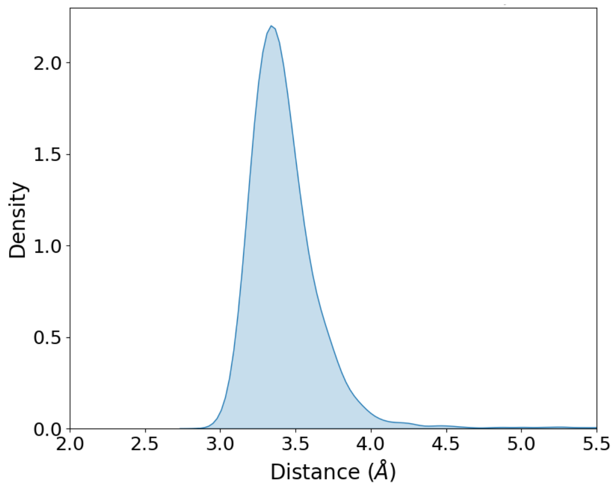

Figure S6. Probability density for the distance between the Asn62Nδ and the Gly167C during the simulation of the Michaelis complex.

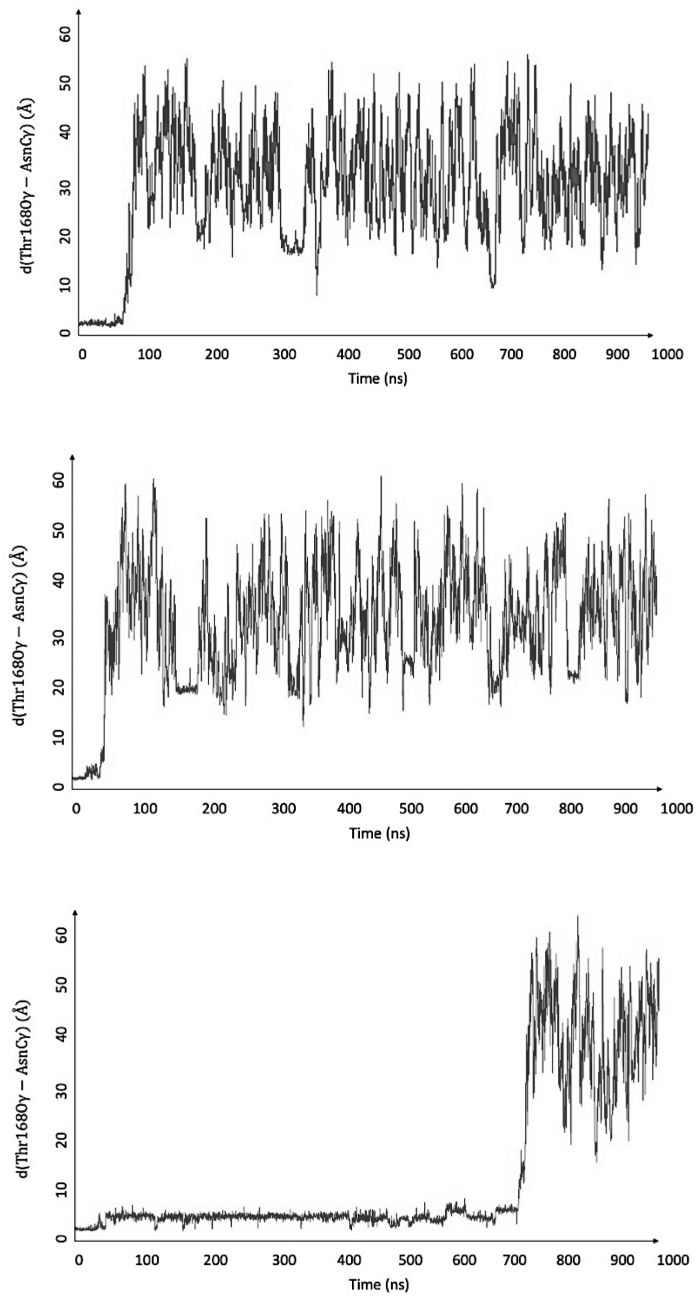

Figure S7. Time evolution of the Thr168Oγ-AsnCγ(Asn) distance in the three replicas of the MD of the Michaelis complex of the monomer form of hASNase3 with substrate (Asn) in the active site

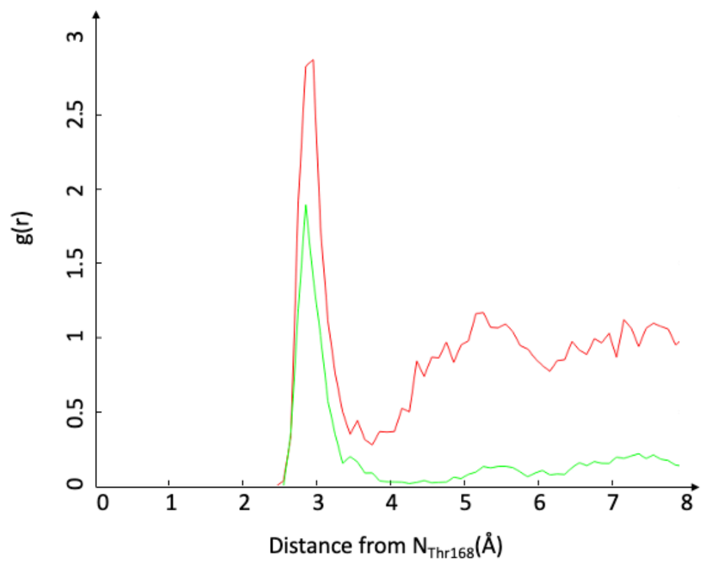

Figure S8. Radial distribution function (RDF) of water environment of Thr168 in the active site of the enzyme (holo form, green line) and a free threonine in solution (red line).

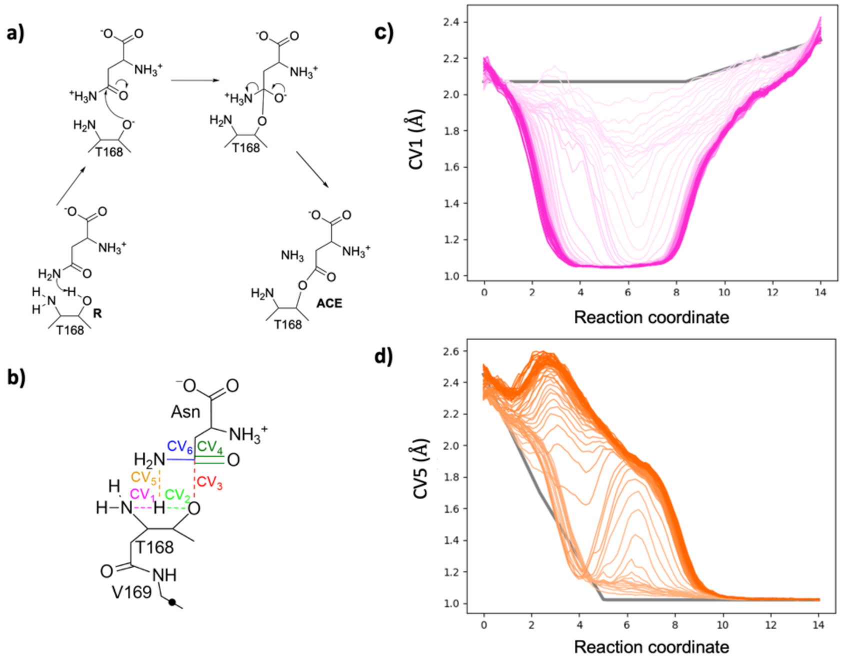

Figure S9. a) Acylation mechanism (formation of acyl-enzyme, ACE) in hASNaseIII where the amino group of the substrate acts as the base activating the nucleophile. b) Representation of the QM region and CVs used to obtain the MFEP. The black dot represents the link atom; c) Evolution of the distance selected as collective variable CV1; d) Evolution of the distance selected as collective variable CV1. Color intensity corresponds to the evolution of the string. Bold grey lines on figures c) and d) indicate the guess values of corresponding CVs along the reaction coordinate. The evolution indicates that the mechanism converges to that presented in Figure 4.

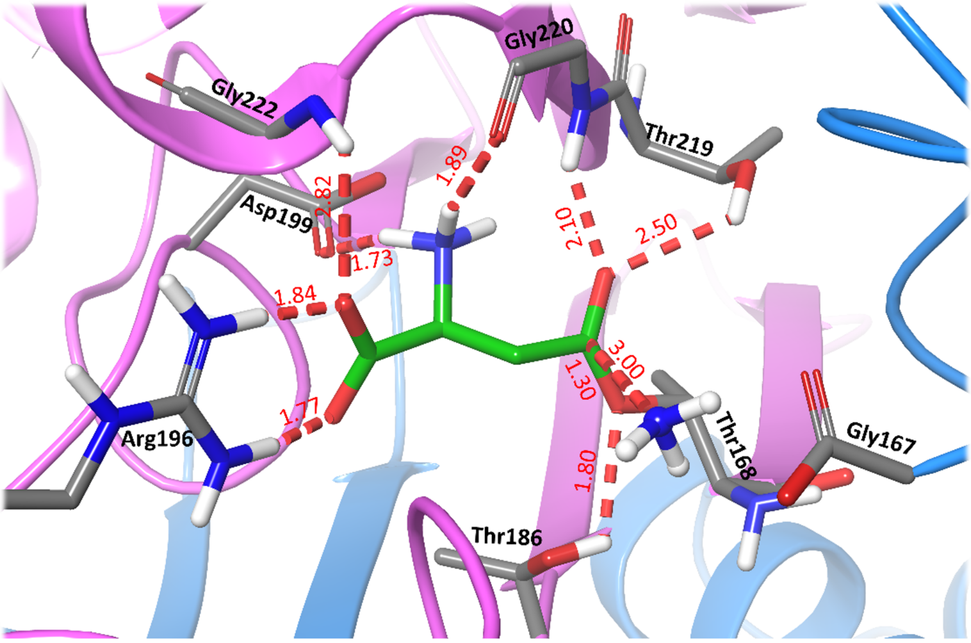

Figure S10. Representation of the active site of the hASNaseIII of the acyl-enzyme product formed (green sticks representations) and ammonia released with the important distances in (Å).

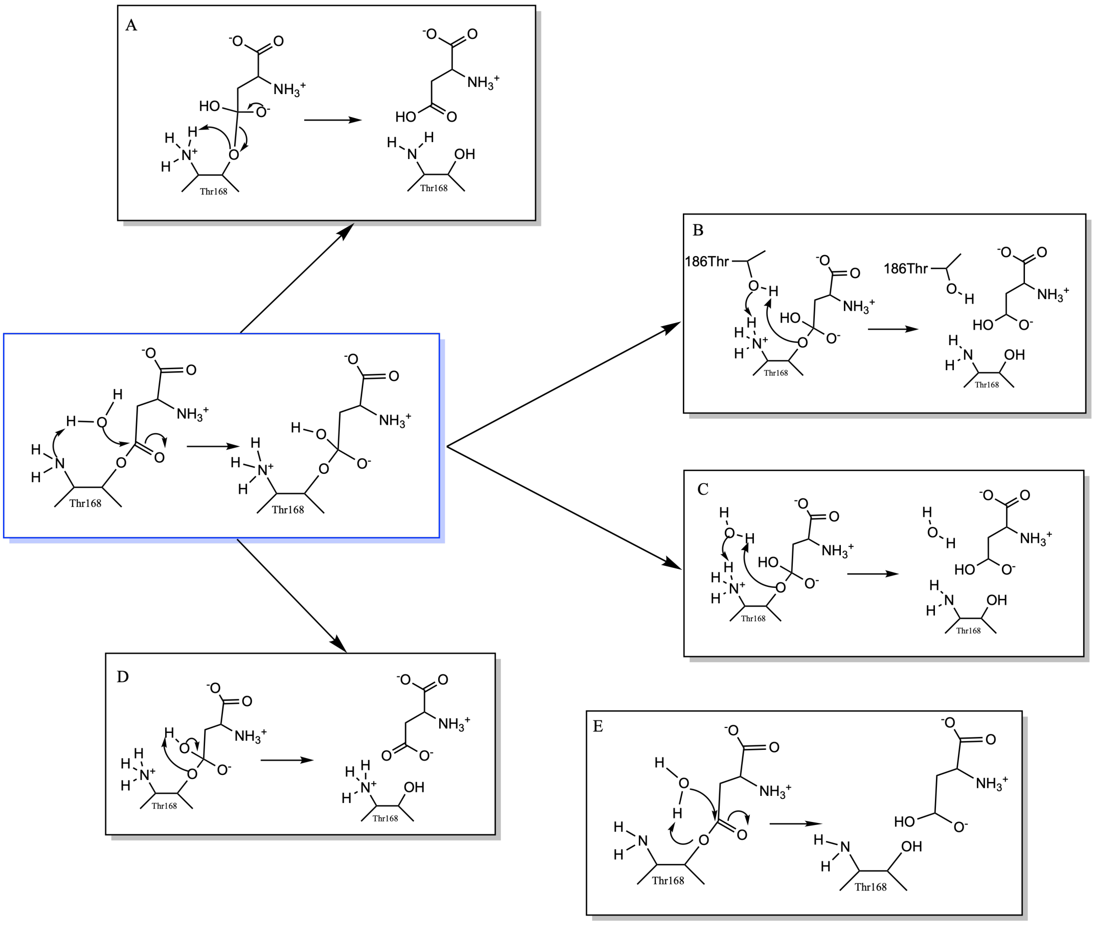

Mechanism	Level of theory	Free energy barrier (kcal·mol[-1])	QM region
A	DFTB3/MM	19.2	ACE + 1wat
	GFN-xTB2/MM	21.6	
	B3LYPD3/6-31+G*	**20.9**	
B	DFTB3/MM	27.9	ACE + 1wat + Thr186 (C_α-C_β)
	GFN-xTB2/MM	/	
	B3LYPD3/6-31+G*	/	
C	DFTB3/MM	21.1	ACE + 2wat
	GFN-xTB2/MM	/	
	B3LYPD3/6-31+G*	/	
D	DFTB3/MM	20.1	ACE + 1wat
	GFN-xTB2/MM	/	
	B3LYPD3/6-31+G*	27.2	
E	DFTB3/MM	Converged to A	ACE + 1wat

Figure S11. Different reaction mechanisms for the acyl-enzyme hydrolisis tested at different levels of theory and the free energy barriers obtained (kcal·mol^{-1}).

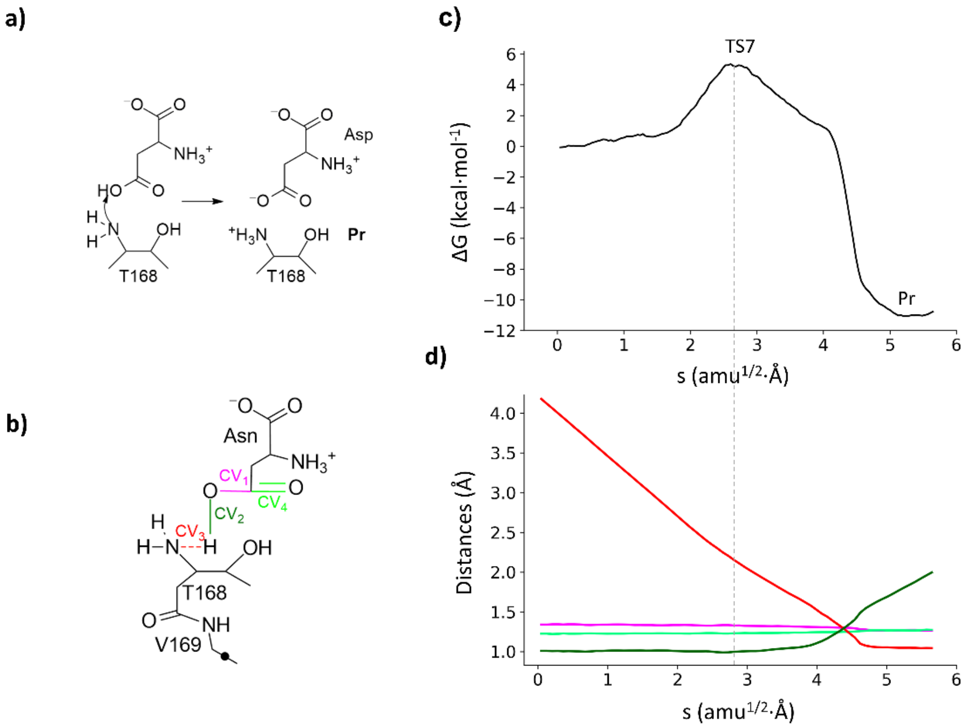

Figure S12. a) Reaction mechanism for regeneration of the hASNaseIII. b) Representation of the QM region and CVs used to obtain the MFEP. The black dot represents the link atom; c) Free energy profile along the path-CV (s) obtained at the B3LYPD3/6-31+G(d)/MM level of theory; d) Evolution of the collective variables along the MFEP. The color code corresponds to that represented in panel b. Dotted light-grey lines indicate the position of the transition states.

Table S5. Free energies (in kcal·mol^{-1}) of the stationary structures corresponding to the PMF for the regeneration stage and corresponding distances found along the MFEP (in Å).

	R	TS7	Pr
Free energy	0.0	5.2	-11.2
CV1 (AsnCγ-Ow)	1.34	1.33	1.27
CV2 (Hw-Ow)	0.99	1.02	1.90
CV3 (Hw-Thr168Nδ)	4.16	2.28	1.02
CV4 (AsnCγ-AsnOδ)	1.25	1.27	1.27

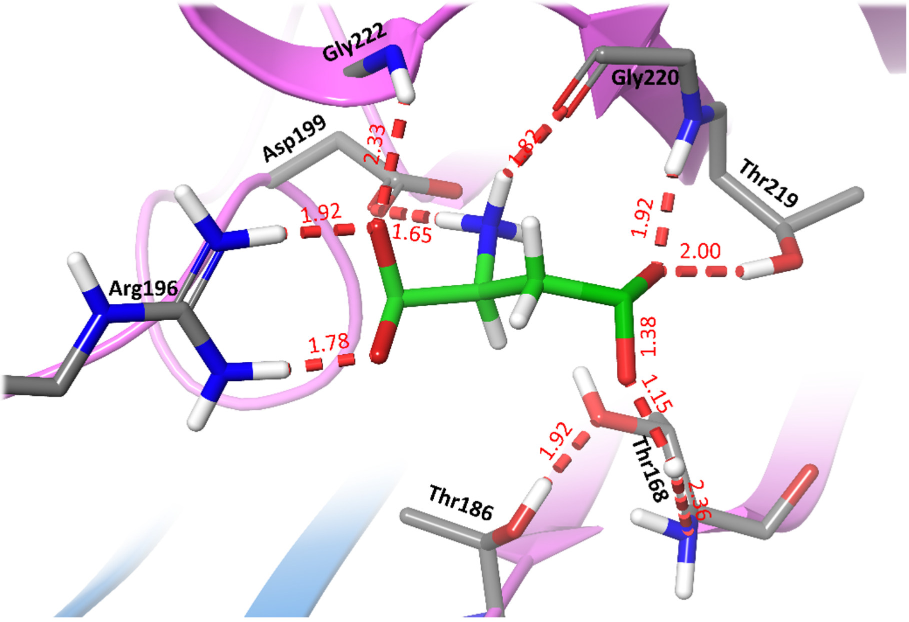

Figure S13. Transition state structure TS7 along with some important distances. All distances are given in Å.

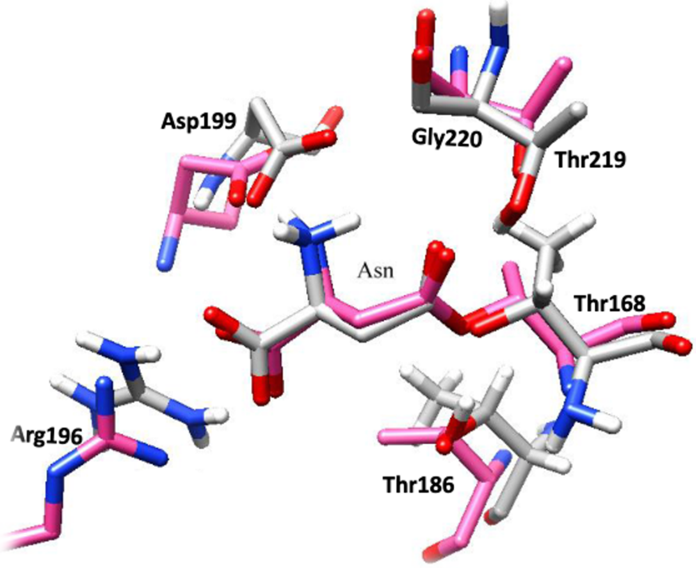

Figure S14. Overlapped structures of active site of X-ray structure (4O0H) of the covalent acyl-enzyme (pink color) and product structure obtained from ASM simulations (grey color).

Video S1. Movie of the mechanism for the formation of the acyl-enzyme complex

Video S2. Movie of the mechanism for the hydrolysis of the acyl-enzyme complex.

Data availability

https://github.com/emedio/hASNaseIII/
The github repository contains all the input files used in MD simulations, including QM/MM ASM simulations. It also includes parameter files (prmtop) of the dimer form of the protein with protonated N-terminal and unprotonated N-terminal. PDB structures of all the transition states along the thermodynamic cycle are provided as well. Repository also contains video animations of the acylation and hydrolysis step of the thermodynamic cycle.

References

(1) Ryckaert, J.-P.; Ciccotti, G.; Berendsen, H. J. C. Numerical Integration of the Cartesian Equations of Motion of a System with Constraints: Molecular Dynamics of n-Alkanes. *J. Comput. Phys.* **1977**, *23* (3), 327–341. https://doi.org/10.1016/0021-9991(77)90098-5.

(2) Le Grand, S.; Götz, A. W.; Walker, R. C. SPFP: Speed without Compromise - A Mixed Precision Model for GPU Accelerated Molecular Dynamics Simulations. *Comput. Phys. Commun.* **2013**, *184* (2), 374–380. https://doi.org/10.1016/j.cpc.2012.09.022.

(3) Salomon-Ferrer, R.; Götz, A. W.; Poole, D.; Le Grand, S.; Walker, R. C. Routine Microsecond Molecular Dynamics Simulations with AMBER on GPUs. 2. Explicit Solvent Particle Mesh Ewald. *J. Chem. Theory Comput.* **2013**, *9* (9), 3878–3888. https://doi.org/10.1021/ct400314y.

(4) Case, D. A.; Ben-Shalom, I. Y.; Brozell, S. R.; Cerutti, D. S.; Cheatham III, T. E.; Cruzeiro, V. W. D.; Darden, T. A.; Duke, R. E.; Ghoreishi, D.; Gilson, H.; Gohlke, H.; Goetz, A. W.; Greene, D.; Harris, R.; Homeyer, N.; Huang, Y.; Izadi, S.; Kovalenko, A.; Kurtzman, T.; Lee, T. S.; LeGrand, S.; Li, P.; Lin, C.; Liu, J.; Luchko, T.; Luo, R.; Mermelstein, D. J.; Merz, K. M.; Miao, Y.; Monard, G.; Nguyen, C.; Nguyen, H.; Omelyan, I.; Onufriev, A.; Pan, F.; Qi, R.; Roe, D. R.; Roitberg, A.; Sagui, S.; Schott-Verdugo, J.; Shen, C. L.; Simmerling, J.; Smith, R.; Salomon-Ferrer, J.; Swails, J.; Walker, R. C.; Wang, J.; Wei, H.; Wolf, R. M.; Wu, X.; Xiao, L.; York, D. M.; Kollman, P. A. AMBER 2018.

(5) He, X.; Liu, S.; Lee, T. S.; Ji, B.; Man, V. H.; York, D. M.; Wang, J. Fast, Accurate, and Reliable Protocols for Routine Calculations of Protein-Ligand Binding Affinities in Drug Design Projects Using AMBER GPU-TI with Ff14SB/GAFF. *ACS Omega* **2020**, *5* (9), 4611–4619. https://doi.org/10.1021/acsomega.9b04233.

(6) Duboué-Dijon, E.; Hénin, J. Building Intuition for Binding Free Energy Calculations: Bound State Definition, Restraints, and Symmetry. *J. Chem. Phys.* **2021**, *154* (20), 204101. https://doi.org/10.1063/5.0046853.

(7) Gallicchio, E.; Levy, R. M. Recent Theoretical and Computational Advances for Modeling Protein–Ligand Binding Affinities. In *Advances in Protein Chemistry and Structural Biology*; Elsevier, 2011; Vol. 85, pp 27–80. https://doi.org/10.1016/B978-0-12-386485-7.00002-8.
